# An Update on the Anticancer Activity of Xanthone Derivatives: A Review

**DOI:** 10.3390/ph14111144

**Published:** 2021-11-11

**Authors:** Yehezkiel Steven Kurniawan, Krisfian Tata Aneka Priyangga, Jumina, Harno Dwi Pranowo, Eti Nurwening Sholikhah, Abdul Karim Zulkarnain, Hana Anisa Fatimi, Jeffry Julianus

**Affiliations:** 1 Department of Chemistry, Faculty of Mathematics and Natural Science, Universitas Gadjah Mada, Yogyakarta 55281, Indonesia; yehezkiel.steven.k@mail.ugm.ac.id (Y.S.K.); krisfian.tata.a@mail.ugm.ac.id (K.T.A.P.); harnodp@ugm.ac.id (H.D.P.); 2 Department of Pharmacology and Therapy, Faculty of Medicine, Public Health and Nursing, Universitas Gadjah Mada, Yogyakarta 55281, Indonesia; etinurweningsholikhah@ugm.ac.id; 3 Department of Pharmaceutical Technology, Faculty of Pharmacy, Universitas Gadjah Mada, Yogyakarta 55281, Indonesia; akarimzk@ugm.ac.id (A.K.Z.); hana.anisa.fatimi@mail.ugm.ac.id (H.A.F.); 4 Department of Pharmaceutical Chemistry, Faculty of Pharmacy, Universitas Sanata Dharma, Yogyakarta 55282, Indonesia; jeffry@usd.ac.id

**Keywords:** xanthone, cancer, in vitro, in vivo, isolation, synthesis, heterocyclic compound

## Abstract

The annual number of cancer deaths continues increasing every day; thus, it is urgent to search for and find active, selective, and efficient anticancer drugs as soon as possible. Among the available anticancer drugs, almost all of them contain heterocyclic moiety in their chemical structure. Xanthone is a heterocyclic compound with a dibenzo-γ-pyrone framework and well-known to have “privileged structures” for anticancer activities against several cancer cell lines. The wide anticancer activity of xanthones is produced by caspase activation, RNA binding, DNA cross-linking, as well as P-gp, kinase, aromatase, and topoisomerase inhibition. This anticancer activity depends on the type, number, and position of the attached functional groups in the xanthone skeleton. This review discusses the recent advances in the anticancer activity of xanthone derivatives, both from natural products isolation and synthesis methods, as the anticancer agent through in vitro, in vivo, and clinical assays.

## 1. Introduction

Cancer is one of the most fatal diseases in the world. Cancer mainly occurs due to gene mutations, and the condition is worsened by other carcinogenic agents. Both gene mutations and carcinogenic agents then influence and change the cell functions and metabolism, and thereby, the replication and spread of cancer cells are uncontrolled. The cancer cells grow and multiplicate rapidly, thus crowding the other normal cells [1]. In some cases, the cancer cells also attack and consume the normal cells, since cancer cells require large amounts of biomaterials for cell division. Because of that, drastic weight loss with lumps on the cancer cell locations is commonly found in cancer patients [2]. In 2020, world cancer cases were reported to be 15 million. It was found that the number of men patients (51%) is slightly higher than the number of women patients (49%). Lung, prostate, and colorectal cancers contribute to 46% of cancer cases in men, while lung, breast, and colorectal cancers contribute to 50% of cancer cases in women [3]. In 2021, it is estimated that around 1.9 million new cancer cases are found in the United States, with around 0.6 million deaths. This means that one in three patients with cancer in the United States do not survive, which is terrifying. The number of cancer and mortality cases has kept increasing, and it is expected that world cancer cases will increase to 23.6 million in 2030 [4].

The research on anticancer drug design and development is an urgent need, because people are suffering and the number of death cases is unstoppable until active, selective, and efficient anticancer drugs are found [5,6,7]. Cancer treatment is one of the most challenging research subjects in the medicinal and pharmacological fields [8,9]. Research into new anticancer drugs from natural sources and synthetic methods is still being conducted. To date, more than 85% of recent bioactive compounds have consisted of heterocyclic structures [10,11]. Among the heterocyclic compounds, hundreds of xanthone derivatives have been isolated, synthesized, and evaluated as anticancer agents [12,13,14,15,16,17,18]. The latest review article on the anticancer activity of xanthone derivatives was reported by Na in 2009 [19]. From that report, unfortunately, an updated review on the anticancer activity of xanthone derivative has not been available yet, as of today. This review, to a certain extent, provides a brief update on the research and development of xanthone derivatives, both from natural products isolation and synthesis methods, such as the anticancer agent through in vitro, in vivo, and clinical assays.

## 2. Xanthone Derivatives as Anticancer Agents

### 2.1. Xanthone Derivatives

**Figure 1:**
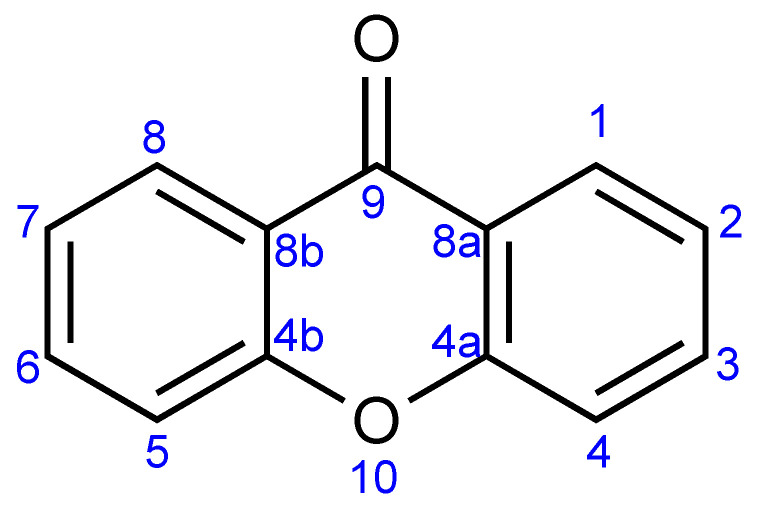
General structure of xanthone.

The name of xanthone was coined by J.C. Roberts in 1961. The word “xanthone” comes from the word for the color yellow, “xanthos” (ξανθός, Greek), since the xanthone compounds are commonly obtained as yellow solids. The first reported xanthone derivative was gentisin, which was isolated from the roots of *Gentiana lutea* in 1821. Xanthone with an IUPAC name of 9*H*-xanthen-9-one is a heterocyclic compound with a dibenzo-γ-pyrone framework, with a basic molecular formula of C_13_H_8_O_2_ [20]. The general structure of xanthone and its atom numbering is shown in Figure 1.

Xanthone is well-known to have “privileged structures” because this simple tricyclic compound exhibits wide biological activities [21,22]. The wide biological activities of xanthone derivatives are caused by their ability to bind with multiple protein receptors. It was reported that xanthone derivatives also exhibit antimicrobial, antidiabetic, antioxidant, antiviral, anti-Alzheimer, anti-inflammatory, and anti-tyrosinase activities. Updates on these biological activities of xanthone derivatives have been available recently [23,24,25,26,27,28,29]. On the other hand, the latest update on the anticancer activity of xanthones was reported in 2009 [19]. The anticancer activity of xanthone derivatives depends on the type, number, and position of the attached functional groups in the xanthone framework. It was reported that xanthone derivatives are able to bind with multiple protein receptors such as cyclooxygenase, protein kinase, and topoisomerase, demonstrating their anticancer activity [22]. These binding properties are also serving in self-monitoring drug application for cancer therapy. In this case, xanthone derivatives act as anticancer agents, as well as sensor agent for selective imaging of cytoplasm of cancer cells through fluorescence spectroscopy [30].

**Figure 2:**
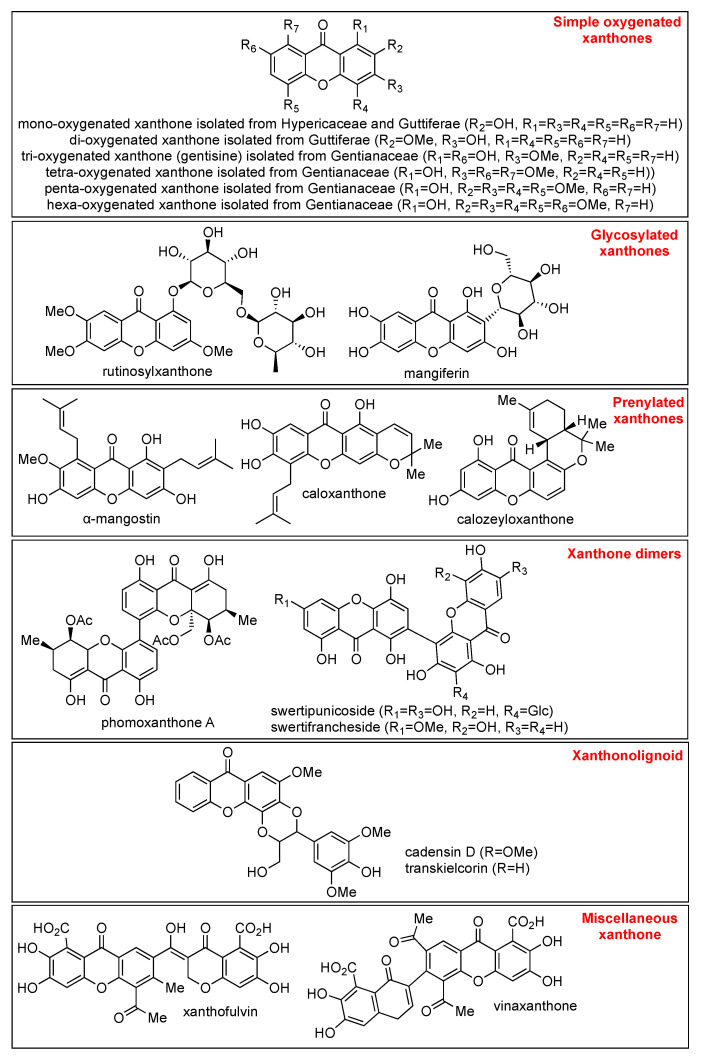
Classification of xanthone derivatives with some examples for each group.

Briefly, xanthone derivatives can be classified into six groups according to their substituents: simple oxygenated xanthone, glycosylated xanthone, prenylated xanthone, xanthone dimers, xanthonolignoid, and miscellaneous xanthone. The simple oxygenated xanthone can be further divided into six subgroups depending on the number of oxygen atoms: mono-oxygenated, di-oxygenated, tri-oxygenated, tetra-oxygenated, penta-oxygenated, and hexa-oxygenated xanthones. The examples of xanthone derivative in each subgroup of oxygenated-xanthone are shown in Figure 2. On the other hand, glycosylated xanthone can be divided into two subgroups: O-glycosides and C-glycosides. In O-glycosides, the glycosidic bond is formed between anomeric carbon atom of sugar ring and oxygen atom of hydroxyl group present in xanthone skeleton. In C-glycosides, xanthone is connected with glycosyl moiety through the C-C bond [31]. The common example of O-glycoside xanthone is rutinosylxanthone, while the example of C-glycoside xanthone is mangiferin, as shown in Figure 2.

The prenylated xanthone group is characterized by the presence of prenyl and geranyl substituents [32]. The prenylated xanthone group contains the largest number of natural xanthone derivatives, such as α-mangostin, caloxanthone, and calozeylxanthone, as shown in Figure 2. Prenyl substituents are being reported as a pivotal functional group for the anticancer activity of xanthone [33]. As an example, Castanheiro et al. reported that the introduction of the prenyl group to the 1-hydroxyxanthone dramatically increased its anticancer activity against the MCF-7 cell line [34]. On the other hand, xanthone dimers or bisxanthones are quite rare, and only around 12 xanthone dimers have been reported so far. The first isolated xanthone dimer was swertipunicoside obtained from the whole plant of *Swertia punicea* in 2002. Swertipunicoside, swertifrancheside, and phomoxanthone A are the examples of xanthone dimers [35], and their chemical structures are shown in Figure 2.

The group of xanthonolignoids is characterized by a connection between xanthone and lignin (coniferyl alcohol) frameworks. The isolated xanthonolignoids have been reported from Guttiferae, such as candesin D and transkielcorin. The number of xantholignoids is also limited. Figure 2 shows the chemical structure of cadensin D and transkielcorin. On the other hand, miscellaneous xanthones are defined for all xanthone derivatives which could not be classified into the other groups, such as xanthofulvin and vinaxanthone, and their chemical structures are shown in Figure 2. Thioxanthones and azaxanthones are also categorized as miscellaneous xanthones. Klein–Junior et al. reported that around 303 simple oxygenated xanthones, 137 glycosylated xanthones, 737 prenylated xanthones, 7 xanthone dimers, 8 xanthonolignoids, and 33 miscellaneous xanthones have been isolated within 2012–2019 [36]. 

### 2.2. Characteristics on Chemical Identification of Xanthone Derivatives

For chemical elucidation purposes, infrared (IR), ultraviolet (UV), mass (MS), and nuclear magnetic resonance (NMR) spectra are required to determine the correct structure of xanthone derivatives [37]. In fact, the IR and UV spectra of xanthone do not serve as accurate tools for structure elucidation. The IR spectrum only recognizes the functional group of xanthones (Table 1); consequently, the position and neighbor conditions of each functional group are not understood well [22]. The UV spectrum of xanthone reflects the absorption characteristics of xanthones chromophore in the ultraviolet region (200–400 nm). The most found chromophore in the xanthone structure is conjugated aromatic and carbonyl moieties; thus, non-chromophore substituents of xanthone are hardly observed. The molecular weight of xanthone can be determined from its MS spectrum. The unsaturation degree of xanthone, as well as halogen substituents, are mostly determined from the MS spectrum. The core structure of the xanthone has one pyran structure, two aromatic rings, and one C=O double bond; thus, the xanthone core itself has an unsaturation degree of 10. The halogen substituent can be indicated by its isotopic effect on the MS spectra. Chloro- and bromo-substituted xanthones commonly give two molecular ion fragments with *m*/*z* of [M]^+^ and [M + 2]^+^ in 3:1 and 1:1 ratios, respectively. In contrast, elucidation of fluoro- and iodo-substituted xanthones from their MS spectrum is difficult, as both halogen atoms are only found as a single isotope. Therefore, fluoro- and bromo-substituents in xanthones can be observed using the ^19^F-NMR and ^127^I-NMR techniques, respectively [38]. 

NMR elucidation is very useful to determine the more detailed structure of xanthone derivatives. The aromatic protons of xanthone are generally found in the chemical shift of 6–9 ppm, depending on the protons’ environment (Table 1). Furthermore, information on the protons’ multiplicity and coupling constant is useful to determine the substituents in the xanthone framework [39]. The nature and position of xanthone substituents can also be evaluated from the ^13^C-NMR spectrum. For the bare structure of xanthone (Figure 1), the xanthone gives 7 carbon signals, as shown in Table 1. The presence of an additional functional group shifts the chemical shift of carbon signals to lower/higher value depending on the nature and location of the functional group [39]. A combination of ^1^H- and ^13^C-NMR of xanthone derivative is also useful for detailed elucidation of xanthone structure. A brief summary on the structure elucidation of xanthones using spectroscopy techniques is shown in Table 1. Other techniques including circular dichroism and X-ray crystallography are very helpful in the final assessment of structure elucidation of xanthone derivatives [39,40,41].

### 2.3. Isolation of Xanthone Derivatives

As of today, thousands of natural xanthone derivatives have been isolated from Amaranthaceae, Anacardiaceae, Annonaceae, Asteraceae, Clusiaceae, Eriocaulaceae, Fabaceae, Filicineae, Gentianaceae, Guttiferae, Hypericaeae, Moraceae, Leguminosae, Loganiaceae, and Polygalaceae families [42,43,44,45,46,47,48,49,50,51,52]. Within 2012–2019, as many as 1225 xanthone derivatives have been isolated from natural sources. Among these families, Clusiaceae and Gentianaceae are the most productive plants to give hundreds of xanthone derivatives. From 2012–2019, 48% and 16% of isolated xanthone derivatives have been obtained only from Clusiaceae and Gentianaceae, respectively. The rest of the isolated xanthone derivatives mainly came from Hypericacae (11%), Moraceae (8%), and Polygalaceae (6%) [34]. These herbs have been used as traditional medicine in several countries, such as China, Thailand, India, and Indonesia. As an example, the whole extract of *Swertia chirata* (Gentianaceae) has been used as an antimalarial drug. The radix extract of *Cudrania cochin* (Moraceae) has been used for hepatitis and rheumatic treatment. Additionally, crude extract of *Monnina obtusifolia* (Polygalaceae) has been used as an anticancer and antimicrobial agent as an indigenous medicine [21].

**Figure 3:**
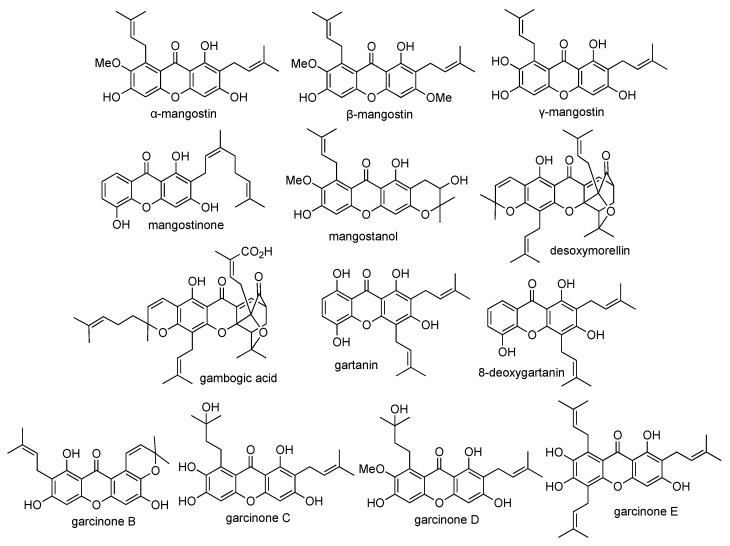
The chemical structure of major xanthones isolated from the pericarp of mangosteen fruit.

*Garcinia mangostana* (Clusiaceae) is a tropical plant that is widely abundant in the Southeast Asia region, such as in Indonesia, Thailand, and Vietnam. One mangosteen plant produces almost 3000 fruits annually. The mangosteen fruit is usually harvested in a spherical form with thin purple peels. The mangosteen fruit is famous as the “queen of fruit” or “superfruit”, due to its multi-biological purposes such as anticancer, anti-inflammatory, antihypertensive, antidiabetic, and antituberculosis activities [37]. In Indonesia, mangosteen fruit has been employed for the traditional treatment of diabetes, hypertension, and cancer. Meanwhile, mangosteen fruit has been utilized for the traditional treatment of fatigue, diarrhea, skin infection, and urinary disorders in Vietnam and Thailand. The mangosteen fruit contains carbohydrates (15%), protein (0.5%), fat (0.4%), and fiber (5.0%) [37]. It was reported that the mangosteen fruit consists of some secondary metabolites, such as xanthones, anthocyanins, flavonoids, phenolics, and organic acids [53]. Remarkably, more than sixty xanthone derivatives have been identified from the pericarp of the mangosteen fruit. The major xanthones isolated from the pericarp of mangosteen fruit are α-mangostin, β-mangostin, γ-mangostin, mangostinone, mangostanol, desoxymorellin, gambogic acid, gartanin, 8-deoxygartanin, garcinone B, garcinone C, garcinone D, and garcinone E. Their chemical structures are shown in Figure 3.

**Figure 4:**
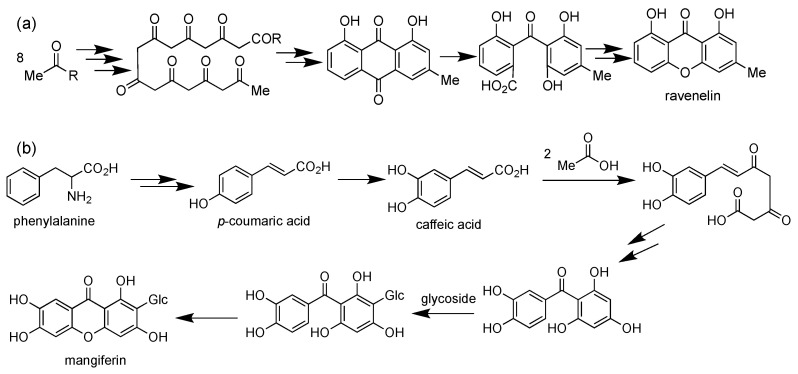
Brief biosynthesis pathway of xanthone derivatives through (**a**) acetate polymalonic pathway (biosynthesis of ravenelin), and (**b**) mixed shikimic acetate pathway (biosynthesis of mangiferin).

The natural xanthone derivatives are mainly generated from acetate polymalonic and mixed shikimic acetate pathways. Birch et al. studied the acetate polymalonic pathway in the biosynthesis of ravelenin from *Helminthosporium ravenelii* using carbon-isotopes labeling [54] (Figure 4a). Eight acyl groups were connected with each other to form 1,3,5,7,9,11,13,15-octaketonic intermediate. Then, the octaketonic intermediate was cyclized to form benzoquinone and benzophenone intermediates. The benzophenone intermediate was further transformed to produce ravenelin. The second pathway (mixed shikimic acetate pathway) was reported by Fujita and Inoue in 1980 for mangiferin biosynthesis in *Anemarrhena asphodeloides* (Figure 4b) [55]. In general, one aromatic ring of xanthone was generated from the shikimic acid pathway, with another aromatic ring coming from the acetate-malonate polyketide pathway. This mixed pathway is responsible for the existence of almost all simple oxygenated and prenylated xanthone derivatives in nature. It was reported that mangiferin was produced from caffeic acid and two acetate units. Glycosidation reaction of mangiferin was found to occur on the benzophenone intermediate. Similarly, the prenylation reaction also happened on the benzophenone intermediate, and not directly on the caffeic acid one. Then, the benzophenone intermediate underwent the ring-closing reaction to form the xanthone as the final product [56]. 

Besides isolation from the terrestrial ecosystem, xanthone isolations from the marine ecosystem have been also reported [57]. The marine ecosystem covers almost 70% of the earth surface, which is equivalent to 95% of the biosphere. Therefore, recent research is focused on the exploration of marine natural products, since the marine natural products may not be found in the terrestrial ecosystem. More than 25,000 marine natural products have been isolated and elucidated, together with hundreds of new marine natural products which have been discovered as of today [38]. Marine fungi have been reported as the most abundant source for xanthone derivatives [58]. 

Several research studies on the isolation of xanthone derivatives from both terrestrial and marine ecosystems are selectively highlighted in this review article based on their anticancer activity report. An anticancer agent can be divided into four classes based on its half-maximal inhibitory concentration (IC_50_) value, i.e., strong (IC_50_ < 5 µM), moderate (IC_50_ 5–10 µM), weak (IC_50_ 10–50 µM), and non-active (IC_50_ > 50 µM) [59]. In 2002, Malmstrom et al. reported the isolation of shamixanthone, tajixanthone and varixanthone from the fungus *Emericella vaericolor*; however, these compounds show no cytotoxic activity against HT29, A549, and P388 cell lines at 1 µg/mL concentration [60]. In 2008, Shao et al. successfully isolated three xanthone derivatives, named as 8-methoxycarbonyl-1-hydroxy-9-oxo-9*H*-xanthene-3-carboxylic acid, dimethyl 8-methoxy-9-oxo-9*H*-xanthene-1,6-dicarboxylate, and methyl 8-hydroxy-6-methyl-9-oxo-9*H*-xanthene-1-carboxylate from fungus *Penicillium* sp. strain ZZF 32#. The crude extracts of these isolated xanthones exhibit cytotoxic activity against KB and KBv200 cell lines with IC_50_ values of 1.50 and 2.50 µg/mL, respectively [61]. 

In 2010, Lee et al. isolated sterigmatocystin and dihydrosterigmatocysin from the fungus *Aspergillus versicolor*. Both compounds exhibited cytotoxic activity against HCT-15, SK-OV-3, A549, XF-498, and SK-MEL-2 cell lines. It was found that sterigmatocystin gave higher toxicity than dihydrosterigmatocysin in an IC_50_ range of 3.76–14.2 µM for these cell lines. Furthermore, sterigmatocystin also gave higher anticancer activity than dihydrosterigmatocysin against Bel-7402 and NCI-H460 cell lines. The structural difference between sterigmatocystin and dihydrosterigmatocysin was the presence of allyl group at the C-5 position of xanthone, demonstrating that the allyl group was critical for anticancer activity [62]. In the same year, Huang et al. successfully isolated two xanthones, named 1,7-dihydroxy-2-methoxy-3-(3-methylbut-2-enyl)-9*H*-xanthen-9-one and 1-hydroxy-4,7-dimethoxy-6-(3-oxobutyl)-9*H*-xanthen-9-one, from the fungus *Avicennia marina*. These isolated compounds exhibited moderate cytotoxic activity against KB and KBv200 cell lines. The 1,7-dihydroxy-2-methoxy-3-(3-methylbut-2-enyl)-9*H*-xanthen-9-one gave IC_50_ value of 20.0 and 30.0 µM, while 1-hydroxy-4,7-dimethoxy-6-(3-oxobutyl)-9*H*-xanthen-9-one gave IC_50_ value of 35.0 and 41.0 µM against KB and KBv200 cancer cell lines, respectively [63].

In 2011, Xu et al. reported the isolation of paeciloxanthone from the fungus *Paecilomyces* sp. This compound exhibited moderate to strong anticancer activity against HepG2 and AChE (acetylcholine esterase) with IC_50_ values of 3.33 and 6.94 µM, respectively. They also reported the successful isolation of secalonic acid D from the fungus *Penicillum oxalicum* and *Paecilomyces* sp. strain ZSU44. The secalonic acid D compound exhibited remarkable anticancer activity against K562 and HL60 leukemia cancer cell lines with IC_50_ values of 0.43 and 0.38 µM, respectively. Furthermore, secalonic acid D was able to inhibit human topoisomerase I enzyme, with an IC_50_ value of 0.16 µM and minimum inhibitory concentration (MIC) value of 0.40 µM. From a further bioassay, it was found that secalonic acid D influenced the GSK-3β/β-catenin/c-Myc pathway, thereby arresting the cancer cell cycle and inhibiting the proliferation of cancer cells [64]. In 2016, Tang et al. reported the isolation of 10 new xanthone derivatives from the leaf of *Garcinia oligantha*. These isolated xanthones belong to dihydroxanthone and tetrahydroxanthone groups. These compounds exhibited moderate to strong anticancer activity against A549, HepG2, HT-29, PC-3, and HL-7702, with IC_50_ values of 3.90–5.50, 4.50–10.0, 4.10–6.40, 3.20–4.60, and 6.40–10.0 µM, respectively [65]. 

In 2017, a novel prenylated xanthone was isolated from the pericarp of *Garcinia mangostana* by Yang et al. This novel xanthone gave moderate to strong anticancer activity against U-87, SGC-7901, PC-3, H490, A549, CNE-1, and CNE-2 cancer cell lines, with IC_50_ values of 6.39, 8.09, 6.21, 7.84, 4.84, 3.35, and 4.01 µM, respectively. The mechanism of action of this novel prenylated xanthone occurred through apoptosis induction on the cancer cells [66]. In 2018, Wei et al. reported the isolation of ananixanthone from the stem bark of *Calophyllum teysmanni*. Furthermore, the isolated ananixanthone was modified through esterification and alkylation reactions to give monoacetate-, diacetate-, 5-methoxy-, and 5-O-benzyl-derivatives. These ananixanthone derivatives exhibited anticancer activity against LS174T, SNU-1, and K562 cancer cell lines, with IC_50_ values in the range of 2.96–50.0 µg/mL. It was reported that methoxy substituents on 5-methoxyananixanthone had higher anticancer activity (14.7 µM) than original hydroxy substituents in the parent compound (19.8 µM). Meanwhile, the diacetate-ananixanthone derivatives gave lower anticancer activity (50.7–119 µM) than the parent compound (7.82–23.7 µM) [67]. In 2019, Kaennakam et al. reported the isolation of two new xanthones, i.e., schomburgones A and B from the bark of *Garcinia schomburgkiana*. Unfortunately, these compounds are non-active against HepG2, MCF-7, HT-29, HeLa S-3, and KB cancer cells, with IC_50_ values of 45.05–69.22 µM [68]. 

**Figure 5:**
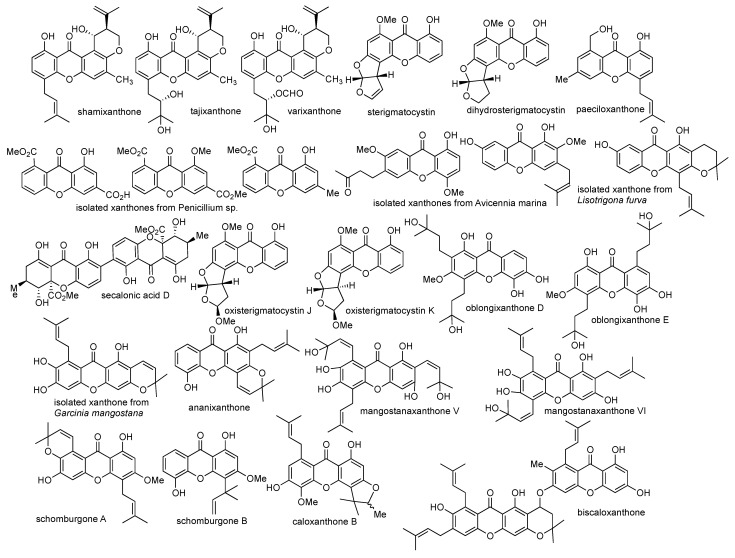
The chemical structure of isolated xanthone derivatives from natural sources.

In 2019, Zamakshshari et al. successfully isolated ananixanthone and caloxanthone B from the stem bark of Calophyllum species. The first isolation of caloxanthone B from Calophyllum species was recorded from this research. Both ananixanthone and caloxanthone B compounds gave moderate and strong anticancer activity against the K562 cell line, with IC_50_ values of 7.21 and 3.00 µM, respectively. The anticancer activity of both compounds may be generated from the protein kinases inhibition [69]. In 2021, Oanh et al. reported another new xanthone derivative isolated from propolis of the stingless bee *Lisotrigona furva*, with weak anticancer activity against SK-LU-1, HepG2, and KB cancer cells in an IC_50_ range of 12.63–34.23 µg/mL [70]. In the same year, Wang et al. reported two new xanthone derivatives: oxisterigmatocystins J and K from fungus *Penicillium* sp. strain DWS10-P-6. Both oxisterigmatocystins exhibited weak anticancer activity against PC-3, MDA-MB-231, and HL-60 cancer cell lines in the IC_50_ range of 12.0–50.0 µM [71]. The chemical structures of the aforementioned xanthones are shown in Figure 5, while the summary of the anticancer activity of isolated xanthone derivatives is listed in Table 2.

The greatest obstacle to the anticancer activity evaluation of isolated xanthones is the very low isolation yield, since xanthones exist as the secondary metabolites in natural sources. To obtain a higher amount of isolated xanthone, the plant parts are cultured and incubated in a certain malt-agar media with tryptone, glucose, and phosphate buffer media, to maintain the pH value. Next, the xanthone derivatives are commonly isolated through a solid-phase extraction using aqueous acetone in 4:1 *v*/*v* as the maceration solvent. Afterwards, the aqueous solvent is subjected to a liquid–liquid extraction with ethyl acetate. Both extracts are then evaporated under vacuum for a further partition process with petroleum ether and/or methanol [38]. 

After the crude extracts have been obtained, the desired xanthones were purified using column chromatography with gradient elution, using a mixture of solvents as the mobile phase. Sephadex LH-20^®^, RP-C18, and silica gel columns are widely used as stationary phase for this purification purpose. In general, xanthone glycosides are isolated using cyanosilane-silica gel and methanol-water-acetonitrile as the solid and mobile phases, respectively. Meanwhile, xanthone aglycones are generally isolated using RP-C18 and phosphoric acid-water-acetonitrile as the solid and mobile phases, respectively. Preparative silica gel chromatography is commonly used for rapid and simple purification of xanthones, however, it gives a less purity than column chromatography [37].

### 2.4. Synthesis of Xanthone Derivatives

As the isolation of xanthone derivatives is complicated and time consuming, the synthesis of xanthone derivatives has been extensively studied. The oldest synthetic route was reported by Kostanecki in 1829. In this synthesis route, the mixture of *ortho*-hydroxybenzoic acid and polyphenol compounds in an equimolar amount was heated in the presence of acetic anhydride or zinc(II) chloride (ZnCl_2_) as the dehydrating agent [72]. Later in 1955, Grover et al. employed a mixture of ZnCl_2_ and phosphorus oxychloride (POCl_3_) as the dehydrating agent at lower temperature, giving a higher yield than the Michael-Kostanecki route. However, the limitation of the Grover route is its incapacity to produce xanthone derivatives from resorcinol and pyrogallol. This inability is caused by the reaction stopping at the benzophenone intermediate [73].

The Grover Shah and Shah (GSS) method is another traditional method to synthesize xanthone derivatives through the cyclodehydration of 2,2′-dihydroxybenzophenone or cycloacylation of 2-aryloxybenzoic acid synthetic route. The mixture of ZnCl_2_ and POCl_3_ is commonly used as the cyclization agent in the GSS method. When the salicylic acid derivative is used as the starting material, then the synthetic route occurs through Friedel–Crafts acylation to form the 2,2′-dihydroxybenzophenone intermediate. The 2,2′-dihydroxybenzophenone intermediate is thus cyclized through a dehydration process to form the xanthone skeleton [74]. On the other hand, when *ortho*-halogen substituted benzoic acid is used as the starting material, then the synthetic route occurs through Ullman condensation to form the 2-aryloxybenzoic acid intermediate. The 2-aryloxybenzoic acid intermediate is thus cyclized through an electrophilic cycloacylation process to form the xanthone skeleton [75].

It was reported that the yield of the GSS synthetic route through benzophenone intermediate is slightly higher than the aryloxybenzoic acid intermediate. The average yield for synthetic xanthone through the GSS-benzophenone route is 60–90%. To improve the synthesis yield, other cyclization reagents have been evaluated, such as a mixture of phosphorus pentoxide (P_2_O_5_) and methanesulfonic acid (MeSO_3_H), trifluoromethanesulfonic acid (F_3_CSO_3_H), trifluoroacetic acid anhydride (F_3_CCO_2_COCF_3_), stannic chloride (SnCl_4_), triphenylphosphine (PPh_3_) in carbon tetrachloride (CCl_4_), and others. So far, a mixture of P_2_O_5_ and MeSO_3_H, named as Eaton’s reagent, gave a remarkable yield (80–95%) for xanthone derivatives. Employing Eaton’s reagent leads to a direct cyclization process of xanthone derivatives with no detectable benzophenone intermediate [74].

Other synthetic methods to obtain a higher yield of xanthones in fewer synthetic steps have been evaluated. Cyclization using F_3_CSO_3_H for diaryl ether is useful to prepare benzoxanthone and/or xanthone dimers in a single-step reaction. Friedel–Crafts acylation followed by the cyclization of 2,2′-dialkoxybenzophenone has been reported as a successful synthesis route of di-oxygenated xanthone. The Robinson–Nishikawa route employs resorcinol and cyanobenzene derivatives to form ketimine intermediate. Afterwards, strong base such as NaOH was employed to hydrolyze the imine functional group and form the ether bond between two aromatic rings. The Tanase synthetic route utilizes salicylaldehyde and phloroglucinol to form the pyran ring. Then, benzylic carbon was further oxidized by chromium(VI) oxide to obtain the xanthone structure. Potassium permanganate on manganese(IV) dioxide or ruthenium(IV) complex can be also used to replace toxic chromium(VI) oxide in the green chemistry approach. The Ullman synthetic route is a successful method to prepare euxanthone from *ortho*-chlorobenzoic acid and phenolic derivatives. The 5,6-dimethylxanthone-4-acetic acid (DMXAA) was successfully synthesized from 3,4-dimethylbenzoic acid in 22% yield using the Ullman synthetic route [76]. 

**Figure 6:**
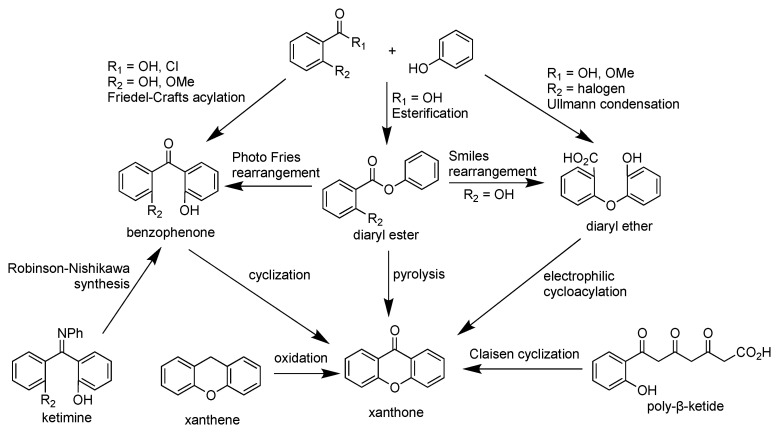
Brief synthetic scheme of xanthone derivatives.

Synthesis of xanthones using 4-picoline or 4-dimethylaminopyridine organocatalyst has been reported from its chromone and acetylene derivatives. This reaction happened through the Diels–Alder cyclization process [77]. Other Diels–Alder approaches are [4+2] cycloaddition of vinylchromone and Fries rearrangement of phenoxyisobenzofuran-1,3-dione with Lewis acid catalyst. The esterification of benzoic acid with phenolic derivative gave diaryl ester as a useful intermediate for the synthesis of xanthone skeleton. The diaryl ester can be further transformed to benzophenone intermediate through Fries rearrangement. On the other hand, the diaryl ester can be converted into diaryl ether intermediate through the Smiles rearrangement. Meanwhile, the direct transformation of diaryl ester to xanthone can be done through the pyrolysis reaction, losing water molecules as the side product. Meanwhile, the Friedländer synthesis is a common route in the synthesis of xanthone glycosides. This reaction occurs between the amino- and carbaldehyde-substituted chromones with ketonic glycosides under alkaline conditions [78]. A brief synthesis scheme of the xanthone derivative is shown in Figure 6.

**Figure 7:**
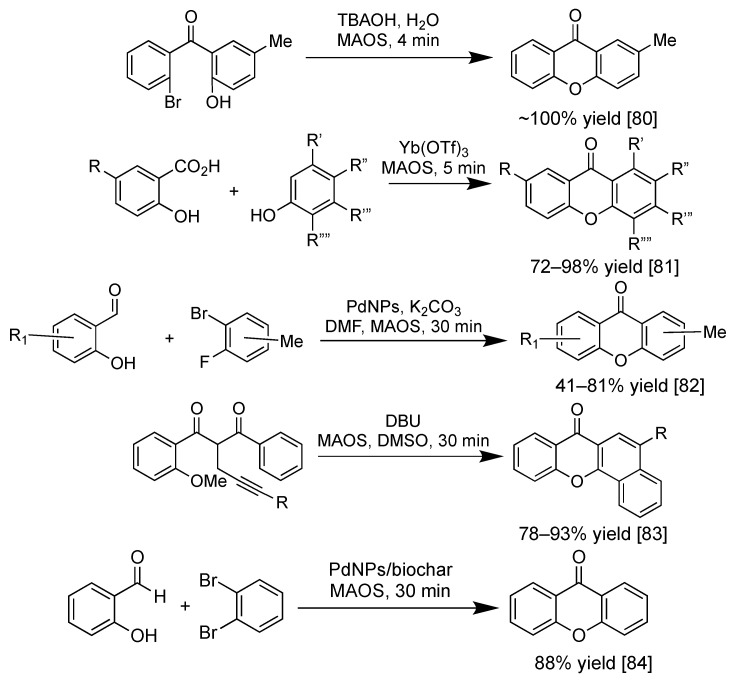
Recent examples of the synthesis of xanthone derivatives using MAOS technique.

Further improvement in the synthesis of xanthone derivatives recently focused on heterogeneous catalysis and microwave-assisted organic synthesis (MAOS). Heterogeneous catalysis is desirable in the green chemistry approach for our sustainable future, as the heterogeneous catalyst material demonstrates a faster synthesis process, a higher product yield, and a milder reaction condition. Moreover, heterogeneous catalyst material can be recovered for further reusability purposes [79]. On the other hand, the MAOS technique has been widely employed in the synthesis of xanthone derivatives, as the required reaction time is significantly shortened, together with dramatic improvement in the product yield and selectivity [80]. The MAOS synthesis of xanthone derivatives from salicylic acid with resorcinol, pyrogallol, cresol, and phloroglucinol gave the corresponding xanthone derivatives in 72–98% yield within 5 min reaction time [81]. Palladium-catalyzed acylation reaction can be used for the formation of xanthone skeleton from salicylaldehyde and dihalotoluene in 41–81% yield [82]. The *tert*-butylammonium hydroxide (TBAOH) as the base has been employed in water-based reaction under MAOS technique to give a quantitative yield of 2-methylxanthone within 4 min [80]. In 2020, metal-free synthesis of benzo[c]xanthone from 1,3-diarylketone was reported by Liang et al., employing 1,8-diazabicyclo[5.4.0]undec-7-ene (DBU) as the base in dimethylsulfoxide (DMSO) solution. The attraction of H-α with DBU leads to the cyclization reaction to form the γ-pyrone scaffold within 30 min in 78–93% yield [83]. Another recent approach to synthesize xanthone derivatives was reported by Steingruber et al., employing salicylaldehyde and dibromobenzene derivatives using palladium nanoparticles. High yield (up to 88% yield), as well as high regioselectivity reaction, were achieved within 30 min reaction time in which the nanopalladium catalyst can be used up to four consecutive cycles without losing its activity [84]. Figure 7 shows the recent examples of the synthesis of xanthone derivatives using MAOS technique.

**Figure 8:**
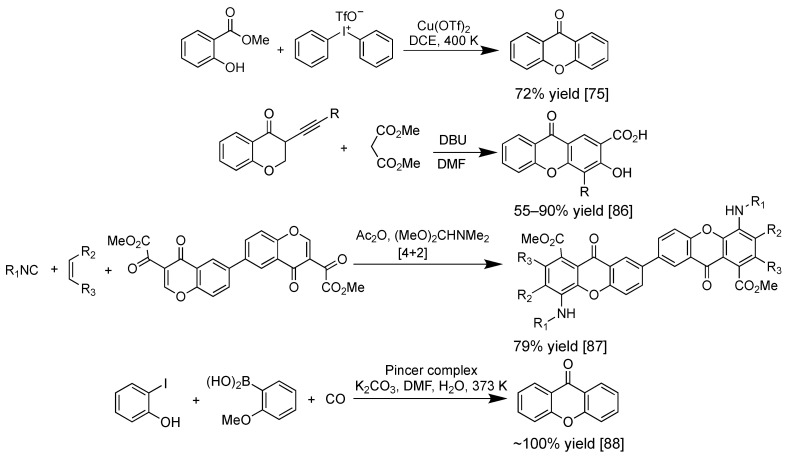
Recent examples of the multireagent synthesis of xanthone derivatives.

Multicomponent reaction in a one-pot synthesis of xanthone derivatives has been attracting researchers’ attention due to the simple and convenient process [85]. In this multicomponent reaction, all the reagents are mixed in a reaction system, sometimes without any usage of solvent, and the final product is the highly substituted xanthone derivative. One-pot synthesis of xanthone was reported by Zhao et al., involving Michael addition, cyclization, 1,2-addition, and elimination reactions in a consecutive process [86]. The coupling reaction between methyl salicylate and diaryliodine triflate salt yielded xanthone derivative in 72% yield, however, the reaction took 12 h to reach the equilibrium state [75]. In 2019, multicomponent synthesis of xanthone dimer was reported from isocyanide, dienophile, and 3-carbonylchromone in 79% yield through [4+2] cycloaddition [87]. Recently, in 2021, one-pot synthesis of xanthone was reported through carbonylative Suzuki coupling reaction by Loureiro et al. This synthetic route utilized three reagents: *ortho*-iodophenol, organoboron, and carbon monoxide, with Pincer palladium as complex as the catalyst material. The reaction yield was quantitative (~100%), however, the reaction was time-consuming (15 h) [88]. Figure 8 shows the recent examples of the multireagent synthesis of xanthone derivatives. More detailed information on the synthesis of xanthone derivatives is available in the published review article by Resende et al. in 2020 [89].

**Figure 9:**
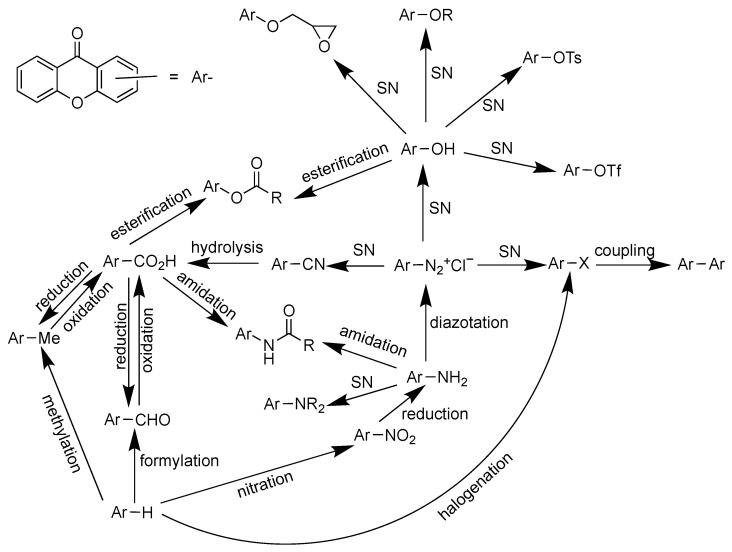
The functional groups’ interconversion of xanthone derivatives.

The functional groups’ interconversion of xanthone derivative is shown in Figure 9. In general, methylation, formylation, nitration, and halogenation reactions of the xanthone aromatic rings can be directly performed. Afterward, the other functional groups can be converted through reduction, oxidation, nucleophilic substitution, esterification, amidation, diazotization, and carbon–carbon coupling reactions. As prenylated xanthones serve as the ideal platform for large biological activities, researchers sometimes do semi-synthesis experiments by introducing a prenyl functional group in the naturally available xanthones. Alkylation of hydroxyxanthone using prenyl bromide with potassium carbonate as the base is the most used protocol to obtain prenylated xanthones. However, the selectivity of this approach is unsatisfying, as the direct alkylation to the aromatic rings of xanthone is inevitable. Thus, this method is only useful for the functionalization of completely substituted xanthones. It means that a selective prenylation of a certain hydroxyl group in the polyhydroxyxanthone is also hardly obtained. For a direct prenylation of the aromatic rings of xanthone, 2-methylbut-3-en-2-ol and boron trifluoride are useful reagents for a selective prenylation reaction. The 1,3-dihydroxy-4-prenylxanthone has been successfully obtained from 1,3-dihydroxyxanthone by using this route. Another approach to obtain prenylated xanthone is through a Claisen rearrangement of prenoxyxanthone. The hydroxyxanthone at first is prenylated using prenylbromide and potassium iodide to obtain the prenoxyxanthone as the intermediate, and then the prenoxyxanthone was heated in the presence of Lewis acid catalyst to obtain the prenylated xanthone. However, it should be kept in mind that further cyclization between hydroxy and prenyl groups sometimes directly occurred to give the dihydrobenzofuran derivative of xanthone [74]. Total synthesis of xanthone derivatives has been reported due to the trace isolation yield of certain xanthones from natural sources. The total synthesis of termicalcicolanone A, α-mangostin, and mangiferin has been reported recently [90,91,92,93].

### 2.5. In Vitro Anticancer Assay of Xanthone Derivatives

**Figure 10:**
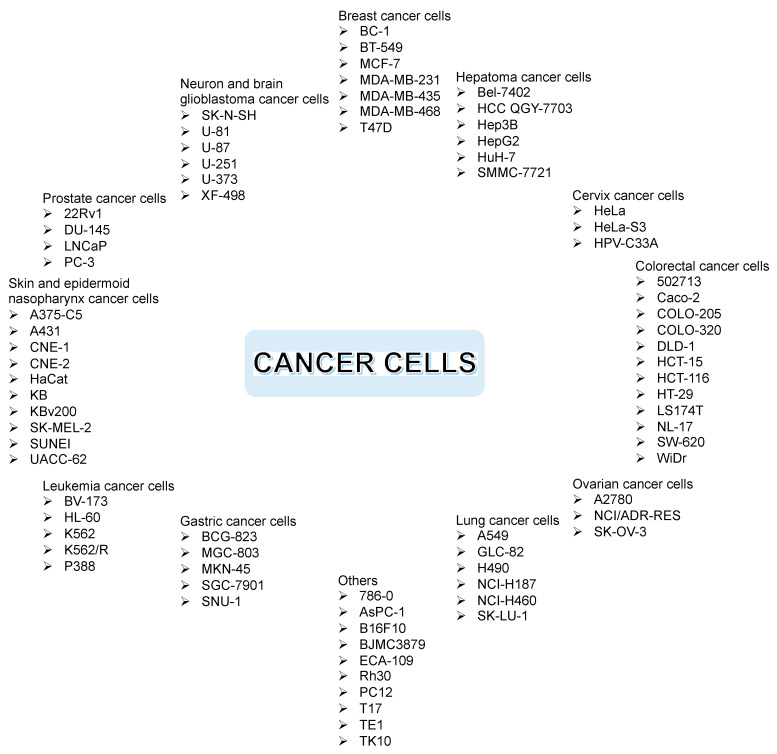
Commonly targeted cancer cells for evaluation through in vitro assay.

Figure 10 shows the commonly evaluated cancer cells through in vitro assay, such as breast, hepatoma, cervix, colorectal, ovarian, lung, gastric, leukemia, skin, epidermoid nasopharynx, prostate, neuron, brain glioblastoma, and other cancer cells, including murine, pancreatic, and renal cancer cells. Each cancer cell has its own characteristics. As an example, NCI-H187 is a small lung cancer cell, while NCI-H460 is a non-small lung cancer cell. As another example, K562 represents sensitive leukemia cancer cells, while K562/R represents drug-resistant leukemia cancer cells. On the other hand, cytotoxic assay for normal cells usually employs H9C2, HEMC, HL-7702, and Vero cell lines [5].

**Figure 11:**
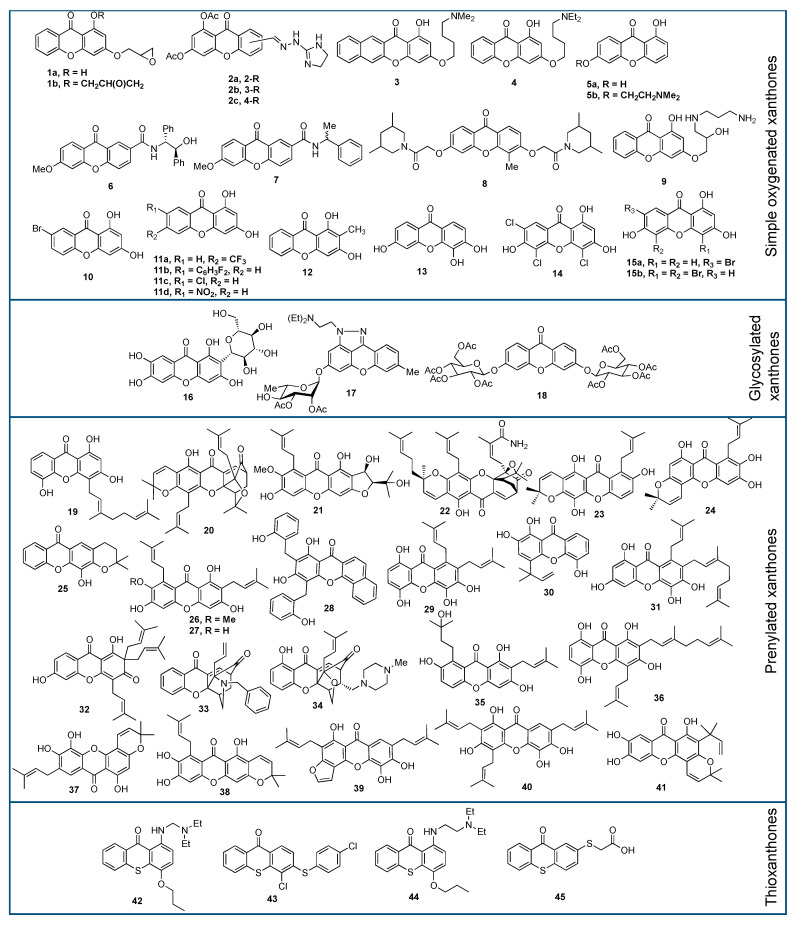
Evaluated xanthone derivatives as the anticancer agents through in vitro assay.

The xanthone derivatives act as anticancer agents through several mechanisms of action. First, activation of caspase proteins induces the apoptosis of cancer cells. Second, inhibition of protein kinases leads to the proliferation of cancer cells. Third, inhibition of aromatase enzyme leads to the inhibition of breast cancer cells’ growth [19]. Fourth, prostaglandin PG-E2 inhibition is another mechanism for the anticancer activity of xanthone derivatives. The PG-E2 is a lipid biomolecule that is involved in the inflammation, angiogenesis, apoptosis, and proliferation of cancer cells [94,95,96,97]. Fifth, topoisomerase inhibition is critical to stop the DNA replication process in cancer cells [98]. Sixth, the inhibition of P-glycoprotein (P-gp) is critical for the multidrug resistance of the cancer cells, since P-gp protein protects the cancer cells by preventing xenobiotics transport into the membrane cells [99,100]. Seventh, RNA bindings and DNA cross-links could significantly suppress the replication of cancer cells [101]. In this section, a brief update on the anticancer assay of xanthone derivatives based on the structure of xanthone derivatives will be described in the following order: simple oxygenated xanthones, glycosylated xanthones, prenylated xanthones, and thioxanthones. The chemical structures of the evaluated xanthones are shown in Figure 11.

Simple oxygenated xanthones are widely explored as anticancer agents. The 1,3-dihydroxyxanthone is well known for its remarkable anticancer activity against cancer cell lines. In 2007, Woo et al. introduced 2-epoxypropyl group on the 1,3-dihydroxyxanthone to improve its inhibitory activity against topoisomerase I and II proteins. Compound **1a** was inactive against MCF-7 and HeLa cells with IC_50_ values of 68.4 and 68.7 µM, respectively. Meanwhile, an additional 2-epoxypropyl group at C-1 position (compound **1b**) drastically improved the IC_50_ values to 3.28 and 23.3 µM against MCF-7 and HeLa cells, respectively [102]. It was also reported that xanthone derivatives with halohydrin, methoxy, and amino substituents at C-3 and C-5 positions were also active for topoisomerase inhibition [103,104]. Furthermore, it was reported that DNA crosslinking occurred on the targeted cancer cells after the treatment with the epoxy-xanthones.

In 2008, Varache-Lembège et al. synthesized several xanthone derivatives and evaluated their anticancer activity. Among the prepared xanthones, compound **2** gave a potential anticancer activity against KB and MCF-7 cell lines through the antiproliferative mechanism. The presence of nitrogen atoms in the heterocyclic rings was found to enhance the anticancer activity of these xanthone derivatives. However, no clear structure–activity relationship was observed with the difference of hydrazonomethyl moiety position. The acetylated group also enhanced the anticancer activity, as the intracellular enzyme could hydrolyze back to dihydroxyxanthone. They found that compound **2a–c** exhibited strong inhibition activity with the IC_50_ values of 2.40, 0.90, and 1.05 µM against KB cells, while these compounds gave IC_50_ values of 1.3, 0.8, and 0.9 µM against MCF-7 cancer cell line, respectively. These values were much lower compared to the doxorubicin with IC_50_ values of 25.0 and 25.7 µM toward KB and MCF-7, respectively [105]. On the other hand, the anticancer activity of synthetic aminoalkoxylated benzo[b]xanthones was evaluated against HepG2, Bel-7402, HeLa, MGC-803, and CNE cell lines. Compound **3** gave the most potent anticancer activity, with IC_50_ values of 3.51, 1.64, 1.59, 0.85, and 0.47 µM against HepG2, Bel-7402, HeLa, MGC-803, and CNE cell lines, respectively. The presence of the dimethylamino group at C-3 position on the benzo[b]xanthone structure seemed to be critical for the anticancer activity against these cell lines [106]. It was reported that aminoalkanol-xanthones could generate the overexpression of manganese superoxide dismutase, thus decreasing reactive oxygen species (ROS)-mediated cell senescence. The aminoalkanol-xanthones also lead mitochondrial dysfunction and cellular apoptosis on the evaluated cancer cell lines [107].

In 2014, Yang et al. examined the anticancer activity of several synthetic compounds based on 1,3-dihydroxyxanthone against CNE, MGC-803, Bel-7402, and A549 cell lines. It was reported that mono- and dioxygen functional groups were pivotal for the inhibitory activity against protein kinases. The presence of the aminoalkoxy group showed the increment of anticancer activity, while the introduction of the bromoalkoxyl group did not improve the anticancer activity. When the terminal amino group was quaternary ammonium salt, the anticancer activity of xanthone became weaker, due to poor cytomembrane penetration. The different terminal amino substituents had different effects in the increment of anticancer activity with the order: diethylamino > dimethylamino > pyrrolidine > piperidine > morpholino. Compound **4** exhibited the best anticancer activity, as measured by inhibitory activity (3.57–20.1 µM) against the aforementioned cancer cells. For a specific MGC-803 cancer cell, compound **4** exhibited a time-and dose-dependent proliferation inhibition. For a concentration greater than 1.0 µM, MGC-803 cells viability decreased significantly, due to lower mitochondrial membrane potential and intracellular calcium leading to the apoptosis mechanism [108].

In 2014, Shen et al. synthesized several xanthones through the modification of the free hydroxyl group of xanthone with dimethylamine group. The inhibitory activity of these xanthones was tested against ECA109, SGC-7901, and GLC-82 cell lines. It was found that the presence of aminoalkyl moiety was critical to its anticancer activity, as well as DNA binding. Moreover, the addition of dimethylamino side-chain resulted in the enhancement of anticancer activity. Compound **5a** gave the IC_50_ value of 25.7, 33.2, and >50.0 µM against ECA109, SGC7901, and GLC-82 cell lines, respectively. Meanwhile, compound **5b** with aminoalkyl moiety gave lower IC_50_ values of 9.56, 13.3, and 16.1 µM against the same cancer cell lines, respectively. The presence of polar aminoalkyl moiety in compound **5b** gave a higher hydrophilicity; thus, this compound could easily penetrate the cancer cell membrane, leading to anticancer activity enhancement. Additionally, the aminoalkyl moiety having a higher pKa value is easily protonated which generate a cationic charge in order to bind with the DNA through its phosphate groups. This phenomenon blocked the DNA replication which is related to the inhibitory activity against the evaluated cancer cell lines [109]. In the same year, Fernandes et al. prepared a chiral xanthone derivative and evaluated its anticancer activity against A375-C5, MCF-7, and NCI-H460 cell lines. Compound **6** was the most potent anticancer agent against the A375-C5, MCF-7, and NCI-H460 cell lines, with IC_50_ values of 32.2, 22.6, and 14.1 µM, respectively. They found that the effect of the growth inhibitory activity of the xanthone compound was not only affected by the nature and position of the substituent but also the enantioselective effect. The presence of aryl group on the stereogenic center of xanthone was found to enhance the inhibitory activity against cancer cell lines [110]. On the other hand, it was reported that (*R*)-isomer of compound **7** gave lower IC_50_ value (IC_50_ = 24.0 µM) than the (*S*)-isomer (IC_50_ = 112 µM) against MCF-7 cancer cell line. This phenomenon was caused by a more stable complex conformation between the (*R*)-isomer with the DNA through crosslinking reactions than the (*S*)-isomer one [111].

In 2016, Liu et al. synthesized xanthone derivatives bearing 3,6-disubstituted aminocarboxymethoxy moiety and evaluated them as the anticancer agent. Among the evaluated xanthones, compound **8** exhibited the highest anticancer activity with the IC_50_ value of 6.18, 8.06, 4.76, 4.59, and 6.09 µM against PC-3, MDA-MB-231, AsPC-1, A549, and HCT-116 cell lines, respectively. Compound **8** promoted cell cycle arrest, induced cell apoptosis, and provided antiproliferative effect on cancer cell viability. The presence of the 4-methyl group was critical in the xanthone structure, as it exhibited a higher potent anticancer activity. In contrast, the presence of oxygen atoms on the side chain of xanthone reduced the growth inhibitory activity against these cancer cell lines. This effect may be generated from the possibility of hydrogen bonds formation with other biological macromolecules outside the active site of the protein target. Additionally, the steric effect of the substituent at the side chain may also contribute to a lower inhibitory activity [112]. A similar result was reported by Dai et al. whereas the substituted phenyl moiety decreased the anticancer activity of xanthone derivatives due to steric effect [113].

Minniti et al. in 2017 investigated the anticancer activity of synthetic xanthone derivatives with various polyamine moieties, including spermine, spermidine, butanediamine, and propanediamine at C-3 position. It was reported that the presence of the secondary amine group in the side chain significantly affected the topoisomerase II-drug interaction. Compound **9** was found to give the most potent inhibition of the catalytic activity of topoisomerase IIα, as evaluated using DNA relaxation assay. Compound **9** inhibited DNA relaxation with the IC_50_ of 1.00 µM. Specifically, compound **9** acts at the DNA cleavage/ligation active side and compound **9** is able to inhibit the ability of DNA to stimulate the rate of ATP hydrolysis. Furthermore, the presence of primary and secondary amine side-chains is more important, rather than the number of primary amines in the chain, or their distance from the primary amine [114].

In 2017, Liu et al. examined the anticancer activity of synthetic xanthones against MCF-7, MDA-MB-231, HepG2, K562, and COLO-320 cell lines. Among the synthesized xanthones, compound **10** showed the highest anticancer activity against MDA-MB-231 cell line, with an IC_50_ value of 0.46 µM. The inhibitory activity of compound **10** against MDA-MB-231 cell line was found to be much stronger compared to DMXAA (IC_50_ = 48.0 µM). Additionally, compound **10** also showed potent inhibitory activity against MCF-7, HepG2, K562, and COLO-320 cell lines, with IC_50_ values of 3.40, 9.20, 13.4, and 10.5 µM, respectively. The inhibitory effect was found as the result of induced apoptosis of the evaluated cancer cell lines. The effect of the electron-withdrawing group introduced to the opposite aromatic ring of 1,3-dihydroxyxanthone derivatives increased the anticancer activity, while the electron-donating group showed the opposite effect. Remarkably, the xanthone derivatives showed an enhanced inhibitory activity when combined with DMXAA. This synergistic effect demonstrated a good approach for the design and development of more potent anticancer drugs [115].

In 2018, Zhou et al. synthesized several xanthone derivatives and evaluated their anticancer activity against A549 and SMMC-7721 cancer cell lines. Compounds **11a**–**b** with fluoro substituents were active against A549 cells, whereas compounds **11c**–**d** were inactive against this cancer cell line. The anticancer activity of compound **11a** (IC_50_ = 29.9 µM) and compound **11b** (IC_50_ = 24.9 µM) was stronger than cisplatin (IC_50_ = 32.4 µM) as the positive control. In contrast, the anticancer activity of non-fluorinated xanthones (IC_50_ = 6.14–14.0 µM) was stronger than that of fluorinated ones (IC_50_ = 27.2–37.4 µM) against SMMC-7721 cell line. The anticancer activity of non-fluorinated xanthones **11c**–**d** (IC_50_ = 6.14–14.0 µM) was also stronger than cisplatin (IC_50_ = 26.3 µM) [116].

Recently, Pedro et al. synthesized oxygenated xanthones and evaluated their anticancer activity against MCF-7, TK-10, and UACC-62 cell lines. Additional oxygenated groups in the xanthone core gave a significant effect on the anticancer activity enhancement. Among several synthesized xanthone derivatives, compound **12** was reported as the best anticancer agent against MCF-7, TK-10, and UACC-62 cells with the IC_50_ values of 21.9, 34.3, and 20.0 µM, respectively. In addition, the compound **12** is considered to be safe for the anticancer application as no cytotoxic to the human lymphocytes (cell viability > 70%) is observed. The nature of substituents in oxygenated xanthone structure had a significant effect on its anticancer activity. For example, the 1,2-dihydroxyxanthone exhibited a weaker anticancer activity than 2-hydroxy-1-methoxyxanthone. Therefore, the suitable functional group should be considered to be a critical parameter in the design of oxygenated xanthone derivative, with high potential for anticancer activity [117].

Our research group has been working on anticancer research employing xanthone derivatives since 2010. Several hydroxyxanthone derivatives including 2-hydroxyxanthone, 3-hydroxyxanthone, 1,3-dihydroxyxanthone, 1,6-dihydroxyxanthone, 3,4-dihydroxyxanthone, 3,6-dihydroxyxanthone, 1,3,6-trihydroxyxanthone, 1,5,6-trihydroxyxanthone, 3,4,6-trihydroxyxanthone, and 1,3,8-trihydroxyxanthone have been synthesized in 2010–2021 [118,119,120,121,122,123,124,125,126]. Among them, 3,4,6-trihydroxyxanthone (**13**) was one of the most active anticancer agents. The anticancer activity of compound **13** against WiDr cells gave an IC_50_ value of 37.8 µM. Furthermore, compound **13** was not toxic against Vero cell lines (IC_50_ = 2510 µM), giving a selectivity index (SI) of 66.4, which was higher than doxorubicin (49.4) [127]. From the quantitative real time-polymerase chain reaction, compound **13** suppressed the mRNA cyclooxygenase-2 (COX-2) expression by 37%, with no inhibitory expression against vascular endothelial growth factor receptors. Therefore, it was concluded that compound **13** inhibited the COX-2 enzyme and started chronic inflammation in the cancer cells. A molecular docking study showed that compound **13** interacted with Tyr355 and Arg120 amino acid residues of COX-2 enzymes, yielding a binding energy of −77.0 kcal/mol [128].

Anticancer activity of chlorinated-hydroxyxanthones has been evaluated against HepG2 and P388 cell lines. Chlorinated-hydroxyxanthones, i.e., 4-chloro-1,3-dihydroxyxanthone, 4,5,7-trichloro-1,3,6-trihydroxyxanthone and 4-chloro-3,6-dihydroxyxanthone are inactive against HepG2 cells with IC_50_ values of 206–666 µM. These IC_50_ values are lower than hydroxyl derivatives (786–828 µM) against the same HepG2 cancer cell line. Furthermore, the SI value of chlorinated-hydroxyxanthones was higher (5.31–22.0) than hydroxyxanthones (2.58–10.60) against HepG2 cells. On the other hand, 4-chloro-1,3-dihydroxyxanthone also gave a lower IC_50_ (12.5 µM) than 1,3-dihydroxyxanthone (68.0 µM) against the P388 cancer cell line. The 4,5,7-trichloro-1,3,6-trihydroxyxanthone gave a lower IC_50_ (5.21 µM) than 1,3,6-trihydroxyxanthone (23.5 µM) against P388 cells. Meanwhile, the 4-chloro-3,6-dihydroxyxanthone gave a lower IC_50_ (0.69 µM) than 3,6-dihydroxyxanthone (10.4 µM) against P388 cancer cells. The SI value of chlorinated-hydroxyxanthones were much higher (87.6–9200) than hydroxyxanthones (31.2–844) against P388 cell line. From the molecular docking study, it was found that chlorinated-hydroxyxanthones were able to inhibit the c-KIT protein through hydrogen bond interactions with Asp810, Cys809, Ile789, His790, and Leu644 [124].

The 4,5,7-trichloro-1,3,6-trihydroxyxanthone (**14**) gave no anticancer activity against HeLa (IC_50_ = 251 µM), T47D (IC_50_ = 1398 µM), and HepG2 (IC_50_ = 262 µM) cancer cell lines. In contrast, compound **14** gave moderate anticancer activity against P388 (IC_50_ = 5.21 µM) cancer cells. Furthermore, the IC_50_ value of compound **14** (IC_50_ = 15.9 µM) was lower than doxorubicin (IC_50_ = 25.4 µM) against Raji lymphoma cell line. The toxicity of compound **14** for Vero cell lines was reported to be in IC_50_ of 256 µM, yielding an SI value of 16.1. From the molecular docking study, compound **14** gave strong binding energy against Raf-1 (−79.4 kcal/mol) and c-Jun-N-terminal protein kinase (c-JNK) (−75.4 kcal/mol) proteins. Compound **14** formed hydrogen bonds with Cys424, Lys431, Ser427, and Gly426. The number of the hydrogen bond interactions (4) of compound **14** with Raf-1 protein was much higher than for the native ligand (1 hydrogen bond with Cys424). Compound **14** generated additional hydrogen bonds with Met111, Glu109, and Ser34 (two hydrogen bonds) on the active site of c-JNK protein. The number of hydrogen bond interactions (4) of compound **14** with c-JNK protein was also higher than the native ligand (2 hydrogen bonds with Met111 and Glu109) [124].

The synergistic effect between compound **14** and doxorubicin against Raji lymphoma cell line has also been studied. It is well-known that drug resistance can be overcome through drug combination as the mechanism of action of two/more drug compounds, which may be different from in their individual usage. Furthermore, a synergistic effect of two/more drugs could lower the dose and thus suppress the side effects to the human body [127]. Combination of both compounds gave the combination index value in a range of 0.06–0.29, indicating a good synergistic anticancer effect. It was reported that compound **14** inhibited Raf-1 protein and activated c-JNK protein. Inhibition of Raf-1 protein led to a higher sensitivity of cancer cells to doxorubicin. On the other hand, the activation of c-JNK protein led to the translocation of pro-apoptotic protein Bax to cytoplasm and stimulated the apoptosis mechanism of cancer cells [128].

Two new brominated-hydroxyxanthones, i.e., compounds **15a** and **15b**, have also been prepared from 2,4-dihydroxybenzoic acid. The compound **15a** was synthesized through bromination reaction of 2,4-dihydroxybenzoic acid first, and then followed by a cyclization reaction with phloroglucinol. On the other hand, compound **15b** was obtained from a bromination reaction of 1,3,6-trihydroxyxanthone. Both compounds gave moderate anticancer activity against P388 cell line, with IC_50_ and SI values of 6.34–10.7 µM and 43.2–74.4, respectively. The molecular docking study revealed that the anticancer activity of brominated-hydroxyxanthones was generated by hydrogen bonding interactions with His790, Cys809, Leu644, Ile789, and Asp810 on the active site of c-KIT protein [129].

Glycosylated xanthones have also been evaluated as anticancer agents. Mangiferin (**16**), a famous glycosylated xanthone, was reported for its anticancer activity against colorectal cancer cells through the inhibition of *bcr/abl* gene expression, thus inducing cellular apoptosis. In 2013, Li et al. isolated mangiferin from mangosteen fruit, and evaluated its anticancer activity against MDA-MB-231 and BT-549 cell lines. Mangiferin exhibited poor anticancer activity against MDA-MB-231 and BT-549 cells, giving the IC_50_ of 299 and 274 μM, respectively. Mangiferin gave a lower expression of matrix metalloproteinase-7 (MMP-7), MMP-9, beta-catenin, and vimentin, and simultaneously gave a higher expression of E-cadherin, thus leading to the antiproliferative phenomenon [130]. On the other hand, mangiferin also acts through influencing cell cycle arrest, activating the caspase-3 protein, and inhibiting the nuclear factor kappa B (NF-κB) pathway [131]. A synergistic cancer treatment employing mangiferin with doxorubicin and oxaliplatin has also been reported to enhance the anticancer activity of mangiferin [132].

In 2015, Song et al. synthesized several xanthone derivatives bearing rhamnopyranoside moiety, and evaluated their anticancer activity against several cancer cell lines. It was found that the presence of sugar moiety was crucial for anticancer activity. Compound **17** exhibited moderate to strong anticancer activity against HL-60, MDA-MB-231, MDA-MB-468, HCT-116, PC3, Rh30, A549, BEL-7402, MKN45, A431, 786-O, and KB cell lines, with IC_50_ values of 2.20, 4.30, 3.47, 2.20, 3.99, 2.37, 1.05, 2.43, 6.29, 2.59, 4.70, and 0.55 µM, respectively. Specific to KB cancer cells, compound **17** could inhibit cell growth by inducing apoptosis, both in extrinsic and intrinsic pathways, and arresting cell cycle progression at the G2/M phase. It was reported that xanthone-O-glycosides at C-3 and C-6 positions were inactive against NCl-H460, MCF-7, and A375-C5 cell lines. However, acylation of all of the hydroxy groups of this xanthone-O-glycosides drastically enhanced the anticancer activity against NCl-H460 and MCF-7 cell lines, with IC_50_ values of 0.19 and 0.46 µM, respectively. This result demonstrated that the acetyl group on the pyranosyl ring was critical for the anticancer activity [133].

In 2019, Alves et al. evaluated the anticancer activity of synthetic xanthone compounds, with acetyl groups against A375-C5, MCF-7, NCI-H460, U-251, U-373, and U-87 cancer cell lines. Compound **18** showed the most potent growth inhibitory activity against these cancer cells. Compound **18** gave IC_50_ values of 135, 0.46, 0.19, 0.55, 0.42, and 0.42 µM against A375-C5, MCF-7, NCI-H460, U-251, U-373, and U-87 cell lines, respectively. The presence of acetyl groups in compound **18** was found to increase its anticancer activity. However, the presence of acetyl groups led to poor water solubility, which can be overcome by encapsulating compound **18** in a drug delivery system [12].

Prenylated xanthones are the most evaluated xanthones, due to their promising application as the anticancer agent. In general, prenylated xanthones exhibited higher anticancer activities than the other classes. Early in 1992, Sordat-Diserens et al. reported that compound **19** isolated from the root bark of *Garcinia livingstonei* gave the IC_50_ value of 1.58 µM against WiDr cancer cells, which was twice as low as 5-fluorouracil (IC_50_ = 3.08 µM) [134]. The anticancer activity of psorospermin has also been reported. Initially, psorospermin was isolated from the radix of *Psorospermum febrifugum* in Africa in 1980. This compound exhibited in vitro and in vivo anticancer activity against wild type and drug-resistant leukemia cells. Furthermore, good anticancer activity of psorospermin against breast and colorectal cancer cells has also been reported. The psorospermin acts as the anticancer agent through DNA intercalation between base pairs at positions 11 and 12, as well as guanine alkylation on the topoisomerase II protein with its epoxydihydrofuran moiety. The DNA alkylation on the active site of topoisomerase II protein leads to inactivation on the DNA replication of cancer cells [135]. Another anticancer agent based on natural xanthone derivatives is desoxymorellin (**20**) isolated from the dry latex of *Garcinia hanburyi*. It was reported that desoxymorellin gave IC_50_ values of 0.74, 0.77, and 1.15 µM against HeLa, K562, and K562/R cell lines, respectively, through the apoptosis induction mechanism [136,137].

In 2006, Laphookhieo et al. isolated a prenylated xanthone from the roots of *Cratoxylum cochinchinense* and evaluated its anticancer activity against NCI-H187. This compound showed a potential cytotoxic effect against NCI-H187 cancer cell line with an IC_50_ value of 1.45 µM. Remarkably, it showed no cytotoxic activity against KB and BC-549 cell lines, which indicated excellent selectivity against NCI-H187 cancer cells. It was proposed that the presence of geranyl moiety in the isolated compound corresponded to its strong anticancer activity [138]. In the same year, Suksamrarn et al. reported the isolation of a new prenylated type xanthone, mangostenone C (**21**), from the early ripe fruit of *Garcinia mangostana*. The anticancer activity of mangostenone C was evaluated against KB, BC-1, and NCI-H187 cells, giving IC_50_ values of 6.11, 7.70, and 8.11 µM, respectively. The anticancer activity of mangostenone C against NCI-H187 was still weaker, but comparable to α-mangostin (5.07 µM) and gartanin (IC_50_ = 2.72 µM). This finding means that the tetraoxygen framework exhibits stronger anticancer activity [139].

Gambogic acid is one of the most famous natural xanthones as an anticancer agent [140,141]. Gambogic acid shows strong anticancer activity against BGC-823, KB, A549, NCI-H460, HepG2, HT-29, MCF-7, DU-145, HL-60, P388, K562/S, and K562/R cell lines in the range IC_50_ of 0.38–4.45 µM [136,142,143,144,145,146]. Furthermore, gambogic acid is very valuable for the treatment of cholangiocarcinoma liver cancer, since no effective anticancer drug is available as of today for this disease [56]. Gambogic acid exhibited anticancer activity for several cancer cells due to many mechanisms of action. The main mechanism of action is apoptosis induction on cancer cells. The apoptosis induction from gambogic acid was generated from several inhibition pathways against NF-microB signaling, c-JNK phosphorylation, G0/G1 phase cell cycle arrest, and Bcl-2 mRNA expression. It was reported that gambogic acid also activated Bax mRNA expression and p53, interacted to transferrin receptor 1 (TfR-1) protein, decreased mitochondria membrane potential, accumulated reactive oxygen species, and depolymerized the microtubules [147,148,149,150,151,152]. These pathways led to a strong apoptosis induction signal on the cancer cells. In 2007, Jang et al. isolated gambogic amide (**22**) as the main component in gamboge, an orange-brownish resin exuded from the plant of *Garcinia hanburryi*, which is often used as traditional medicine for cancer treatment. The anticancer activity of gambogic amide was found to be related to the apoptotic activity against T17 cells with an IC_50_ of 5.00 nM. The T17 cell line was derived from the basal forebrain of SN56 cell; therefore, T17 cells were defined as TrkA stably transfected SN56 cells. The gambogic amide could selectively bind to TrKA, triggering its tyrosine phosphorylation, and preventing neuronal cell death. These phenomena yielded an agonist effect to nerve growth factor (NGF) thus giving potent neurotrophic activity. Moreover, gambogic amide could block apoptotic machinery independent of the Trk receptor, which led to its ability to trigger programmed cell death for the cancer cell lines [153].

Two new xanthones isolated from *Terminalia calcicola* named as termicalcicolanones A (**23**) and B (**24**) have been reported in 2007. Termicalcicolanones A and B showed weak to moderate anticancer activity against A2780 cells, with IC_50_ values of 40.6 and 8.10 µM, respectively [154]. With a similar structure, the positions of hydroxyl and pyranosyl moieties on termicalcicolanone significantly influence anticancer activity [155]. A combination of pyranoxanthone and paclitaxel has also been reported to enhance their anticancer activity [156]. In 2010, Palmeira et al. synthesized xanthone derivatives by modifying the dihydroxyxanthone through prenylation and ring closure reactions. The synthesized compounds were evaluated as the anticancer agents against K-562, HL-60, and BV-173 cell lines. They found that compound **25** gave the most promising inhibitory activity on the K-562 cell viability, with an IC_50_ value of 20.0 µM. Additionally, compound **25** gave a moderate inhibitory activity against HL-60 and BV-173, with an IC_50_ value of 7.00 and 14.0 µM, respectively. The inhibitory mechanism was suggested to come from the antiproliferative and apoptotic effects on the evaluated cancer cell lines [157].

In 2011, Johnson et al. evaluated the anticancer activity of pure α-mangostin (**26**) against LNCaP, 22Rv1, DU 145, and PC-3 cancer cell lines, with IC_50_ values of 5.90, 6.90, 22.5, and 12.7 µM, respectively. The α-mangostin could induce cancer cell apoptosis at a concentration equal to or higher than 15 µM. Meanwhile, α-mangostin can promote G0/G1 cell cycle arrest at a lower concentration (<15 µM) [158]. Other research groups reported that α-mangostin also showed moderate anticancer activities against T47D, MDA-MB-231, PC12, DLD-1, and HL-60 cell lines, with IC_50_ values of 2.44–28.5 µM [159,160,161,162,163,164]. The main mechanisms of action for α-mangostin as the anticancer agent were antiproliferation, apoptosis induction, and dysfunction of mitochondria [161,165]. Furthermore, the other mechanisms of action for α-mangostin were observed through influencing the G0/G1 phase cell cycle arrest and inhibition of tau-phosphorylation, p38 mitogen-activated protein kinase (MAPK), human epidermal growth factor receptor 2/phosphatidylinositol-3-kinase/Akt (HER2/PI3K/Akt), and extracellular signal-regulated protein kinase 1/2 (ERK1/2) signaling pathways [34,166]. A synergistic effect between α-mangostin and cisplatin has been reported for cervix cancer cells treatment [167]. The introduction of prenyl group at C-1 position dramatically increased the anticancer activity of α-mangostin against the MCF-7 cell line [34]. Azevedo et al. explained that 2,2-dimethyl-3,4-dihydropyran moiety was critical for the anticancer activity against HL-60, A375-C5, NCl-H460, and MCF-7 cells [168]. On the other hand, the hydroxyl groups at C-3 and C-6 positions were also critical for the anticancer activity of α-mangostin against B16F10, MDA-MB-231, AsPC-1, SW-620, and NCI-H460 cell lines [169].

In 2012, Chang and Yang isolated another natural xanthone, γ-mangostin (**27**), from the hull of mangosteen fruit. The γ-mangostin was one of the major xanthone components in the mangosteen fruit. The γ-mangostin was evaluated as the anticancer agent against HT-29 cells. Unfortunately, γ-mangostin was non-active against HT-29 cells (IC_50_ = 68.5 µM). This inhibitory activity occurred through the induced apoptosis mechanism [170]. Cheng et al. synthesized benzoxanthones and evaluated their anticancer activity against A549, MDA-MB-435, and HCT-116 cell lines. Compound **28** showed the most potent anticancer activity, with IC_50_ values of 14.3, 15.8, and 5.17 µM for A549, MDA-MB-435, and HCT-116, respectively. The inhibitory activity of compound **28** as an anticancer agent relies on topoisomerase I protein inhibition, which led to the DNA relaxation of cancer cells [171].

In 2012, Niu et al. isolated a bioactive compound-based xanthone derivative from the stem bark of *Garcinia bracteata* and evaluated its anticancer activity against HL-60. The extraction, partition, and purification of the stem bark of *Garcinia bracteata* yielded up to 31 xanthone derivatives. Among them, three compounds: compound **29**, globuxanthone (**30**), and garciniaxanthone E (**31**) exhibited strong anticancer activity against HL-60 cell line, with IC_50_ values of 2.80, 3.40, and 3.10 µM, respectively. Compound **29** and garciniaxanthone E had prenyl moiety at their structure which corresponded to a stronger anticancer activity against HL-60 cells, indicating that the prenyl groups play an important role in the growth inhibitory activity. The number of prenyl groups was also suggested to significantly increase the inhibitory activity of xanthone. However, the addition of prenyl groups at the side-chain as in garciniaxanthone E did not significantly affect its activity compared to compound **29**. Garciniaxanthone E was also reported for its anticancer activity against breast, lung, liver, gastric, colorectal, and leukemia cancer cell lines through the activation of caspase proteins and inhibition of PG-E2 pathways [172].

Lim et al., 2012 synthesized several xanthone derivatives owing to the prenyl group, and evaluated their anticancer activity against HeLa and MDA-MB-231 cell lines. Among the synthesized compounds, compound **32** showed the most promising anticancer activity. Compound **32** gave IC_50_ values of 8.90 and 4.50 µM against HeLa and MDA-MB-231 cell lines, respectively. The anticancer activity of compound **32** was twice as strong as doxorubicin and cisplatin as the positive standards. The presence of nonplanar geminal-diprenylated rings was suggested to enhance its inhibitory activity against the cancer cell lines [173]. In the same year, Zhang et al. synthesized several xanthone derivatives which belong to the aza-caged Garcinia analogues. The synthesized compounds were evaluated as anticancer agents against HepG2, A549, and U-251 cell lines. Among them, compound **33** showed the highest anticancer activity, with IC_50_ values of 2.62, 2.10, and 16.4 µM against HepG2, A549, and U-251 cell lines, respectively. Moreover, compound **33** also showed inhibitory activity against serine/threonine protein kinase, nuclear factor kappa-B kinase subunit gamma (IKK-β), with an IC_50_ value of 8.02 µM. The introduction of hydrophobic moiety in the aza-caged xanthone structure leads to a stronger anticancer activity. Moreover, it was reported that the presence of substituent group with larger volume was preferred in the anticancer activity enhancement, due to preferable interaction with the putative receptors. Compound **33** exhibited the anticancer activity through the apoptosis induction mechanism [174].

In 2013, Zhang et al. synthesized a natural xanthone derived from Garcinia species and evaluated its anticancer activity against HepG2 and A549 cell lines. Compound **34** was considered to give strong anticancer activity against HepG2 and A549 cell lines with IC_50_ values of 3.25 and 3.60 µM, respectively. Furthermore, compound **34** was also found to have good bioavailability, and it was orally active, as tested through an in vivo assay. Compound **34** was capable of giving the inhibition rate of 58% against tumor growth (hepatoma solidity/Heps) at 100 mg/kg daily oral dose for 4 days, which was much better compared to the natural product with a similar structure, gambogic acid. However, a further clinical evaluation has not been reported yet [175].

In 2015, several isolated xanthones from the branches of *Garcinia achachairu* were investigated as the anticancer agents against nasopharynx cancer cells (CNE-1, CNE-2, and SUNE1). A new prenylated xanthone (**35**) showed a remarkable anticancer activity with the IC_50_ of 1.43, 0.73, and 2.23 µM against CNE-1, CNE-2, and SUNE1 cell lines, respectively. On the other hand, the anticancer activity of compound **36** was evaluated against U-251, MCF-7, NCI/ADR-RES, 786-0, NCI-H460, PC-3, HT-29, and HaCat cell lines, giving IC_50_ values of 11.2, 34.0, 8.61, 19.6, 56.2, 10.5, 58.1, and 30.6 µM, respectively, which was comparable to doxorubicin [176]. The mechanism of anticancer activity of both compounds was suggested through the antiproliferative effect on the evaluated cancer cell lines [177]. In 2016, Li et al. isolated xanthone derivatives from the leaves of *Garcinia paucinervis* plant, which yielded a new compound, paucinervin I (**37**). Paucinervin I showed strong anticancer activity against HL-60 cell line with the IC_50_ value of 1.30 µM. The anticancer activity of paucinervin I was stronger than 5-fluorouracil (IC_50_ = 2.37 µM). The presence of angular pyranoxanthone skeleton in paucinervin I compound seemed to play a pivotal role in its antiproliferative activity [178]. In 2017, Yang et al. isolated a new prenylated xanthone derivative, 7-O-demethyl mangostanin (**38**), from the pericarp of mangosteen fruits, and studied its anticancer activity against CNE-1, CNE-2, A549, H490, PC-3, SGC-7901, and U-87 cancer cell lines. The 7-O-demethyl mangostanin gave IC_50_ values of 3.35, 4.01, 4.84, 7.84, 6.21, 8.09, and 6.39 µM against CNE-1, CNE-2, A549, H490, PC-3, SGC-7901, and U-87 cancer cell lines, respectively. The 7-O-demethyl mangostanin gave a stronger anticancer activity when compared to the Hirsutanol A as the positive control (IC_50_ = 6.30–15.0 µM). The mechanism of action of 7-O-demethyl mangostanin was generated by the induction of the late and early-stage apoptosis of cancer cells [67].

In 2018, Jia et al. isolated xanthone derivatives from the stem of *Garcinia paucinervis* and evaluated their anticancer activity against HL-60, PC-3, and Caco-2 cell lines. The isolation process yielded two new xanthones, (-)paucinervin O (**39**) and pacinervin P (**40**). Compound **39** showed strong anticancer activity, with IC_50_ values of 0.87 and 2.06 µM against HL-60 and Caco-2 cells, respectively. Compound **39** owing a furan ring was found to be more active against three cancer cell lines compared to the compounds with a dihydrofuran ring. On the other hand, compound **40** gave a strong anticancer activity, with IC_50_ value of 4.66 µM against PC-3 cells [179]. In 2019, Liu et al. synthesized several xanthone derivatives and evaluated their potential anticancer activity against hepatoma cancer cells, i.e., HepG2, Hep3B, SMMC-7721, and HuH-7. They found that the presence of the 3-methyl-2-butenyl group was crucial for the enhancement of anticancer activity specifically against HepG2 cells. Additionally, when 3-methyl-2-butenyl and 1,3-dihydroxy moieties formed a cyclic conjugate system, a much stronger anticancer activity was observed against all evaluated cancer cells. Among them, compound **41** was found to give the strongest anticancer activity, with IC_50_ values of 18.6, 36.5, 52.8, and 69.6 µM against HepG2, Hep3B, SMMC-7721, and HuH-7, respectively. The mechanism of its anticancer activity was reported through interactions with caspase 3, caspase 9, and poly(adenosine diphosphate-ribose) polymerase (PARP) proteins, which participate in programmed cell death [18].

Since a limited number of isolated and synthesized xanthone dimers and xantholignoids are found, both groups are rarely investigated as anticancer agents. On the other hand, thioxanthone is a xanthone derivative in which the oxygen atom of the pyran ring is replaced by a sulfur atom [180,181]. In 2012, Palmeira et al. reported the synthesis and anticancer activity of 1-substituted 4-propoxythioxanthone against K562 cancer cells. It was reported that compound **42** with the (*N*,*N*-diethylamino)ethylamino functional group at C-1 position was the most active anticancer agent. Compound **42** gave IC_50_ value of 1.90 µM, which was much lower than doxorubicin (12.0 µM) [157]. In 2015, Chen et al. synthesized 3-substituted-4-chloro-thioxanthones and evaluated their anticancer activity for several cell lines. These thioxanthone analogues were prepared by Ullmann condensation and Friedel–Crafts intramolecular reactions. It was known that the functional group attachment at C-3 of thioxanthone enhanced its anticancer activity. Among the prepared thioxanthone derivatives, compound **43** showed the most promising anticancer activity. Compound **43** gave moderate to strong anticancer activity, with IC_50_ values of 7.90 and 3.90 µM for MCF-7 and MDA-MB-468 cells, respectively. Compound **43** showed no significant toxicity effect toward H9C2 cells (>25.0 µM), demonstrating that it could be applied as a potential candidate for an anticancer lead compound [182].

**Figure 12:**
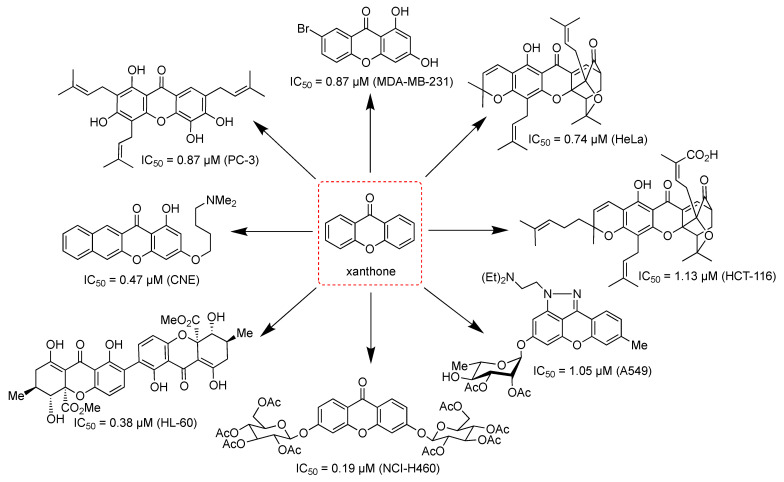
Summary of the most promising anticancer agents based on xanthone derivatives in this review.

In 2016, Barbosa et al. examined the anticancer activity of synthetic thioxanthone derivatives against MCF-7, NCI-H460, and A375-C5 cell lines. Compound **44** showed the most potent anticancer activity, with an IC_50_ value of 6.10, 6.00, and 3.60 µM against MCF-7, NCI-H460, and A375-C5 cells, respectively. The anticancer activity was caused through the induced apoptosis mechanism. The modification of compound **44** to its hydrochloride salt form was found to enhance its solubility and bioavailability [183]. In 2020, Ataci et al. synthesized compound **45** and tested its cytotoxic anticancer activity against HT-29 cells. Compound **45** showed no anticancer activity (IC_50_ > 165 µM) against HT-29 cell lines. In contrast, UV light irradiation stimulated compound **45** to enhance its cytotoxic activity (IC_50_ = 65.5 µM). Moreover, compound **45** exhibited higher activity compared to the common chemotherapeutic agents, such as 5-fluorouracil (IC_50_ = 222 µM) and cisplatin (IC_50_ = 66.4 µM). Under UV light irradiation, compound **45** released CO_2_ and produced alkyl radicals, thus increasing its anticancer activity. Therefore, compound **45** has potential for its application as a theragnostic agent toward specific targeted cancer treatment [184]. The summary of the in vitro anticancer activity of xanthone derivatives is listed in Table 3. Meanwhile, the summary of the most promising anticancer agents based on xanthone derivatives in this review article is shown in Figure 12.

A molecular docking study of xanthone and thioxanthone derivatives against platelet-derived growth factor response (PDGFR) and epidermal growth factor receptor (EGFR) protein kinases has been evaluated in our previous study. It was found that hydroxyxanthones with halogen substituents gave stronger binding energies (−6.87 to −7.25 kcal/mol) than erlotinib (−6.58 kcal/mol) against EGFR protein. However, they gave weaker binding energies (−7.92 to −8.57 kcal/mol) than imatinib (−13.4 kcal/mol) against PDGFR protein. A similar hydrogen bond of Met769 with erlotinib was also found on the hydroxyxanthones with halogen substituents. Furthermore, the hydroxyxanthones with halogen substituents gave an additional hydrogen bond with Thr766; consequently, they gave stronger binding energy than imatinib against EGFR protein. Meanwhile, a similar hydrogen bond with Cys673 of imatinib was also found on the hydroxyxanthones with halogen substituents. However, the hydrogen bond between imatinib with Asp810 was not found in the hydroxyxanthones with halogen substituents; thus, it may be the reason why they gave weaker binding energy than imatinib against PDGFR protein [185]. On the other hand, the molecular docking study of xanthyl chalcone derivatives has also been conducted against KIT tyrosine kinases. The inhibition of KIT protein kinases led to inhibiting cell growth and proliferation of signal transduction on small cell lung cancer, gastrointestinal stromal, and myeloid leukemia cancer cells. The xanthyl xanthone derivatives gave stronger binding energies (−8.79 to −10.9 kcal/mol) as well as lower inhibition constants (10–364 nM) than sunitinib (−8.25 kcal/mol; 890 nM), due to the presence of hydrogen bonds with Lys593 and Cys673 amino acid residues [186].

Rational design on the structure and anticancer activity of xanthone derivatives has been reported; however, a comprehensive and holistic point of view is still very limited. For example, it was reported that the hydroxyl group at C-1 position of xanthone was critical for the anticancer activity against MCF-7 [117]. The 1-hydroxyxanthone derivative was 6 times more active in the anticancer activity than xanthone with the absence of 1-hydroxyl substituent. On the other hand, it was reported that 1,3-diacetyl groups gave stronger anticancer activity against MCF-7 cells [105]. The introduction of the prenyl group to the 1-hydroxyxanthone was also able to dramatically increase its anticancer activity against MCF-7 cell line [34]. Azevedo et al. stated that diethylamino at C-8 position together with 2,2-dimethyl-3,4-dihydropyran moiety were critical for the anticancer activity of xanthone against MCF-7 cells. On the other hand, the presence of electron-withdrawing group on 1,3-dihydroxyxanthone enhanced anticancer activity [187]. These results are confusing, since each researcher reported based on a limited number of anticancer activities of the isolated and/or synthesized xanthone derivatives.

Quantitative structure–activity relationship (QSAR) is a computational approach to rationalize the relationship between the chemical structure of drugs with their biological activity [188]. A QSAR study on the anticancer activity of 33 haloxanthones against HepG2 and MCF-7 cells was performed in our previous study. Haloxanthones gave anticancer activity against both cancer cells, due to the activation of c-JNK protein through hydrogen bond interactions with Met111 and Gln109, as well as halogen interactions with Met108 and Asp112. From the QSAR analysis, it was found that IC_50_ of haloxanthones against HepG2 cancer cells can be predicted through log(IC_50_) = −1.198(qC7) + 9.274(qC8b) − 1.887(qC8) − 23.35(qO carbonyl) − 6.034 (R^2^ = 0.94), where qX represented the charge of atom X. On the other hand, it was found that IC_50_ of haloxanthones against MCF-7 cancer cells can be predicted through log(IC_50_) = 100(LUMO) + 0.36(DM) − 3.02(qC6) − 1.71(qC4) − 7.98(qC1) + 7.85 (R^2^ = 0.89), whereas LUMO and DM represent the lowest unoccupied molecular orbital and dipole moment, respectively [189]. A QSAR study of xanthone derivatives as topoisomerase IIα inhibitors was reported by Alam and Khan in 2014. It was found that IC_50_ of xanthones as topoisomerase IIα inhibitors can be predicted through pIC_50_ = 2.19(DE) + 0.22(n(OH)) − 0.54(LogP) − 0.469(SIB) + 0.018(SSA) + 2.57 (R^2^ = 0.84), whereas DE, n(OH), LogP, SIB, and SSA represent dielectric energy, the group count of hydroxyl substituents, logarithm of the partition coefficient between *n*-octanol and water, shape index basic (order 3), and solvent-accessible surface area, respectively [190].

A more comprehensive QSAR study on the anticancer activity of xanthone derivatives was investigated by Alam and Khan in 2018 [191]. They evaluated the anticancer activity of more than one hundred xanthone derivatives from Garcinia species and built QSAR mathematic models. They reported that the IC_50_ value of xanthone derivatives against A549 cancer cell line can be predicted through log (IC_50_) = −39.0090(x1) − 0.8078(x2) − 1.0827(x3) − 0.0483(x4) + 0.5053(x5) − 0.0560 (R^2^ = 0.87), whereas x1 is a parameter of electronegativity contribution, x2 is the atom type count descriptor class, x3 is the number of carbon atoms connected with four single bonds, x4 is the number of C=C bonds, and x5 is the electrotopological state of methyl group. Meanwhile, the IC_50_ value of xanthone derivatives against HepG2 cancer cell line can be predicted through log IC_50_ = −0.6407(y1) − 0.0336(y2) − 0.1278(y3) + 0.2226(y4) + 0.5877(y5) + 0.5940 (R^2^ = 0.87), whereas y1 is electrotopological state of carbon atoms, y2 is the number of double bonds, y3 is the number of hydrogen bond acceptor atoms, y4 is electrotopological state of methine group, and y5 is the number of oxygen atoms. On the other hand, the IC_50_ value of xanthone derivatives against U251 cancer cell line can be predicted through log IC_50_ (µM) = 0.0948(z1) + 0.5217(z2) + 0.3687(z3) + 1.1313 (R^2^ = 0.86), whereas z1 is the electrotopological state of nitrogen atoms, z2 is the number of oxygen atoms, and z3 is the topological state of carbon atoms. However, a more comprehensive QSAR study involving all reported xanthone derivatives against a certain cancer cell is not available yet, as of today. Therefore, computational studies on this research are still open to be investigated by researchers in the future.

### 2.6. In Vivo and Clinical Anticancer Assays of Xanthone Derivatives

The xanthone derivatives showed promising in vitro anticancer activity; thus, further investigation through in vivo and then clinical assays is required [168]. The in vivo test of α-mangostin revealed the potential usage of α-mangostin as chemotherapeutic and chemopreventive agents, since it could suppress tumor formation in nude mice induced 22Rv1 cell lines. Oral administration of α-mangostin was able to reduce the growth of 22Rv1 cells by more than 5 times compared to the negative control (without any oral administration of α-mangostin), due to the activation of the caspase-3 protein [158]. It was also reported that oral α-mangostin consumption reduced growth by 50–70% and reduced 40% of the mass of NL-17 cancer cells in the mice by suppressing the cell proliferation and activation of apoptotic mechanisms [192]. A similar result for the oral administration of α-mangostin was also reported to be effective for COLO-205 and HCT-116 cells [193]. Meanwhile, intraperitoneal injection of α-mangostin on the mice inhibited 50% of the growth of GBM8401 due to the increment of adenosine monophosphate-activated protein kinase (AMPK) protein thus triggering the autophagy of cancer cells [194]. On the other hand, γ-mangostin was reported for its ability for HT-29 cells through mitochondrial collapse and apoptosis pathways [170]. An in vivo investigation on mice through oral administration of γ-mangostin was conducted. After 1.5 h, the γ-mangostin compound was mostly found in the blood plasma of mice [130].

It was reported that panaxanthone (a mixture of α-mangostin and γ-mangostin in 6:1 wt/wt) inhibited the DNA replication and induced the G1 phase cell cycle arrest of cancer cells [195]. Subcutaneous injection of panaxanthone significantly inhibited the metastatic growth of BJMC3879 cancer cells in the mice. The inhibitory activity was related to the activation of caspase-3 and caspase-9 proteins, with a loss of cytochrome c from the mitochondria causing the collapse of the mitochondria membrane potential of cancer cells [196]. On the other hand, subcutaneous injection xanthone extract from *Garcinia mangostana* fruit rinds containing 81% of α-mangostin and 16% of γ-mangostin significantly reduced the tumor volume of HCT-116 on mice. The inhibitory activity was related to the induction of apoptosis and inhibition of cancer cells migration, invasion, and clonogenicity [197].

A clinical study for mangosteen juice (containing 3.90 g/L of α-mangostin) to the human group (20 people) was conducted [198]. It was found that maximum α-mangostin concentration was up to 3.10 µg/L, while the time to maximum plasma concentration (T_max_) was found to be at 1 h, with a decrement to 65% after 4 h. A similar result was reported in China for maximum α-mangostin concentration (4.20 µg/L) and T_max_ (1 h) value in the human group (20 people) [199]. In 2012, Chitchumroonchokchai and coworkers reported the clinical study of mangosteen juice (containing 1.22 g/L of α-mangostin) to the human group (10 people) with a high-fat breakfast. It was found that the maximum α-mangostin concentration was up to 0.45 µg/L, while the T_max_ value was found to be at 2–4 h on average [200]. Faster distribution of α-mangostin can be achieved through intravenous administration. It was reported that, after intravenous injection, the α-mangostin was metabolized to form in its hydrogenated, oxidized, glucuronidated, and methylated forms [201].

Detailed anticancer investigation on DMXAA has also been reported. The DMXAA leads to vascular collapse and tumor necrosis through tumor necrosis factor-α induction. Synergistic anticancer activity with chemotherapy agents such as doxorubicin, imatinib, erlotinib, and sunitinib, as well as with the cancer radiotherapy technique has been reported. DMXAA exhibited low toxicity to the normal cells with suitable pharmacokinetic parameters. Furthermore, the clinical trials of DMXAA as the anticancer agent have been evaluated in New Zealand and the United Kingdom. The phase I clinical trial of DMXAA to 63 patients gave no drug-related myelosuppression. The DMXAA was well tolerated up to 4900 mg/m^2^ dose. At a higher dose, the DMXAA gave the side effects of visual disturbance, tremors, confusion, slurred speech, and anxiety [202]. Unfortunately, the DMXAA only gives strict species-specificity to the mouse stimulator of interferon genes (STING), with no significant activity against human STING in phase III clinical trial. Researchers are doing their best to modify the chemical structure of DMXAA, to obtain more active anticancer agents such as in alkylated-, alkoxylated-, fluorinated-, hydrazonated-, cyclic-, and heterocyclic substituted-derivatives [203]. However, no significant improvement in anticancer activity has been achieved yet.

**Figure 13:**
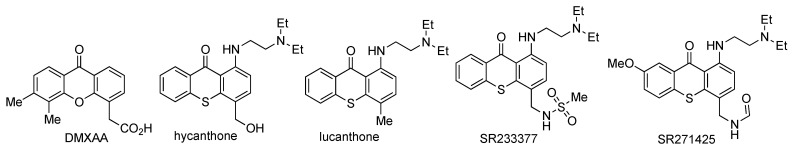
Evaluated xanthone derivatives as the anticancer agents through in vitro assay.

As an isostere of xanthone, thioxanthones have similar structures and physicochemical properties to xanthone derivatives [204]. Nowadays, dozens of thioxanthone derivatives are under investigation for a possible application as anticancer agents [99,205]. Hycanthone and lucanthone are anticancer drug candidates based on thioxanthone structure [206]. Their anticancer activity was related to the topoisomerase inhibition and DNA intercalation mechanisms. However, their clinical trials were stopped due to their mutagenic effect. Other potential anticancer agents based on thioxanthones are SR233377 and SSR271425. However, their clinical trials were also withdrawn due to their cardiotoxic effect [207,208,209]. The chemical structures of DMXAA, hycanthone, lucanthone, SR233377, and SSR271425 are shown in Figure 13.

The most serious drawbacks for the utilization of xanthone derivatives as the anticancer agent are their poor solubility in water and their reactivity to be transformed into phase 2 conjugates form. Therefore, further technologies are critical to overcome these limitations [210,211]. It was reported that the coating of gambogic acid in the form of nanoparticles core shell dramatically increased its anticancer activity against HepG2 and A549 cancer cells due to the intracellular drug delivery process [212]. To overcome the poor solubility of thioxanthone, Yilmaz et al. prepared polymeric thioxanthone materials and evaluated their anticancer activity against A549 cells in the radiotherapy process. Radiotherapy is sometimes used to enhance the activity of anticancer agents through ionizing radiation. It was reported that ionizing radiation acts as a sensitizer to enhance the inhibition of cancer cells proliferation. The thioxanthone derivative was connected to polyvinyl alcohol and polyethylene glycol materials through covalent bonds. Two types of polymer materials were prepared: thioxanthone-polyethylene glycol through C-N amide bond, and thioxanthone-polyvinyl alcohol through C-O acetal bond. The thioxanthone-polyvinyl alcohol did not decrease the A549 cell viability percentage at 0.03 mg/mL. In contrast, thioxanthone-polyethylene glycol decreased the A549 cell viability percentage to 70% at the same concentration. It means that thioxanthone-polyethylene glycol gave a higher anticancer activity against A549 cancer cells than thioxanthone-polyvinyl alcohol. Radiating the thioxanthone-polyethylene glycol material with 2.5 Gy exposure drastically decreased the A549 cell viability percentage to 30%. This research offers an option for the usage of low solubility xanthone and/or thioxanthone derivatives for in vivo and clinical uses [213]. In contrast to the reports on the clinical study of α-mangostin and DMXAA that are available now, the reports on both in vivo and clinical study of other xanthone derivatives are very limited. Therefore, extensive efforts are still needed for the development of the anticancer drug for our better health and future.

## 3. Conclusions and Future Direction

This review highlights the potential anticancer of xanthone derivatives, contributing to the design and develop of new drugs. Both isolated and synthesized xanthone derivatives have been proven to have promising anticancer activities against breast, hepatoma, cervix, colorectal, ovarian, lung, gastric, leukemia, skin, epidermoid nasopharynx, prostate, neuron, brain glioblastoma, and other cancer cells through in vitro assay. However, their further evaluation as anticancer agents through in vivo and clinical assays is still limited as of today. Furthermore, a comprehensive QSAR study for the better design of anticancer drugs based on the xanthone structure has not yet been established. These research gaps require extensive collaborations and contributions from researchers around the world to further develop active, selective, and efficient anticancer drugs based on xanthone derivatives.

## Figures and Tables

**Table 1:** A brief summary on the structure elucidation of xanthones using spectroscopy techniques.

Xanthone Structure	IR (cm^−1^)	UV (nm)	^1^H-NMR (ppm)	^13^C-NMR (ppm)
Csp^2^-H	3000–3100	-	6–9	-
C=O	1650–1720	200–400	-	176
C=C aromatic	1450–1600		-	126 (C-1)
				124 (C-2)
				135 (C-3)
				118 (C-4)
				156 (C-4a)
				121 (C-8a)
C-O-C ether	1000–1200	-	-	-
O-H	3300–3500	-	9–10 (C-2 or C-7)	-
			9–11 (C-4 or C-5)	
			10–11 (C-3 or C-6)	
			12–14 (C-1 or C-8)	

**Table 2:** Summary of the anticancer activity of isolated xanthone derivatives.

Xanthone Derivative	Source	IC_50_ (Cancer Cells)	Ref.
Crude extract of xanthones	Fungus *Penicillium* sp. strain ZZF 32#.	1.50 µg/mL (KB)	[61]
		2.50 µg/mL (KBv200)	
Sterigmatocystin	Fungus *Aspergillus versicolor*	3.76 µM (SK-MEL-2)	[62]
1,7-Dihydroxy-2-methoxy-3-(3-methylbut-2-enyl)-9*H*-xanthen-9-one	Fungus *Avicennia marina*	20 µM (KB)	[63]
		30 µM (KBv200)	
1-Hydroxy-4,7-dimethoxy-6-(3-oxobutyl)-9*H*-xanthen-9-one		35 µM (KB)	
		41 µM (KBv200)	
Secalonic acid D	Fungus *Penicillum oxalicum*	0.43 µM (K562)	[64]
		0.38 µM (HL60)	
Dihydroxanthone	Leaf of *Garcinia oligantha*	3.90 µM (A549)	[65]
		3.20 µM (PC-3)	
Tetrahydroxanthone		5.50 µM (A549)	
		4.60 µM (PC-3)	
Prenylated xanthone	Pericarp of *Garcinia mangostana*	3.35 µM (CNE-1)	[66]
		4.01 µM (CNE-2)	
5-methoxyananixanthone	Stem bark of *Calophyllum teysmanni*	14.7 µM (K562)	[67]
schomburgones A	Bark of *Garcinia schomburgkiana*	45.05 µM (HepG2)	[68]
		52.21 µM (HeLa S-3)	
Ananixanthone	Stem bark of Calophyllum species	7.21 µM (K562)	[69]
Caloxanthone B		3.00 µM (K562)	
Isolated xanthone	Propolis of the stingless bee *Lisotrigona furva*	12.63 µg/mL (HepG2)	[70]
		14.36 µg/mL (SK-LU-1)	
Oxisterigmatocystins J	Fungus *Penicillium* sp. strain DWS10-P-6	15.14 µM (HL-60)	[71]
Oxisterigmatocystins K		21.62 µM (MDA-MB-231)	
		12.06 µM (HL-60)	

**Table 3:** Summary of the in vitro anticancer activity assay of reported xanthone derivatives.

Xanthone Derivative	Source	IC_50_ (µM) (Cancer Cells)	Main Mechanism	Ref.
**1a**	Synthesis	68.4 (MCF-7)	Topoisomerase inhibition, DNA crosslinking	[102]
**1b**		3.28 (MCF-7)		
**2a**	Synthesis	1.3 (MCF-7)	-	[105]
**2b**		0.8 (MCF-7)		
**2c**		1.05 (KB)		
**3**	Synthesis	0.47 (CNE)	Mitochondrial dysfunction	[106]
**4**	Synthesis	3.57 (MGC-803)	Mitochondrial dysfunction	[108]
**5a**	Synthesis	25.7 (ECA109)	DNA binding	[109]
**5b**		9.56 (ECA109)		
**6**	Synthesis	22.6 (MCF-7)	DNA crosslinking	[110]
(*R*)-**7**	Synthesis	24.0 (MCF-7)	DNA crosslinking	[111]
(*S*)-**7**		112 (MCF-7)		
**8**	Synthesis	4.59 (A549)	Promoting cell cycle arrest	[112]
**9**	Synthesis	1.00 (-)	Topoisomerase IIα inhibition	[114]
**10**	Synthesis	0.46 (MDA-MB-231)	Apoptosis induction	[115]
**11a**	Synthesis	27.16 (SMMC-7721)	-	[116]
**11b**		24.9 (A549)		
**11c**		6.14 (SMMC-7721)		
**11d**		14.02 (SMMC-7721)		
**12**	Synthesis	20.0 (UACC-62)	-	[117]
**13**	Synthesis	37.8 (WiDr)	Suppressing mRNA COX-2 expression	[127]
**14**	Synthesis	5.21 (P388)	Raf-1 and c-JNK inhibition	[124]
**15a**	Synthesis	6.34 (P388)	c-KIT inhibition	[129]
**15b**		10.7 (P388)		
**16**	Isolation	274 (BT-549)	bcr/abl gene expression inhibition	[130]
**17**	Synthesis	0.19 (NCl-H460)	Promoting cell cycle arrest	[133]
**18**	Synthesis	0.19 (NCI-H460)	-	[12]
**19**	Isolation	1.58 (WiDr)	-	[134]
**20**	Isolation	0.74 (HeLa)	Apoptosis induction	[136]
**21**	Isolation	6.11 (KB)	-	[140]
**22**	Isolation	0.005 (T17)	Apoptosis induction	[153]
**23**	Isolation	40.6 (A2780)	-	[154]
**24**		8.10 (A2780)		
**25**	Synthesis	7.00 (HL-60)	Apoptosis induction	[157]
**26**	Isolation	5.90 (LNCaP)	Apoptosis induction	[158]
**27**	Isolation	68.5 (HT-29)	Apoptosis induction	[170]
**28**	Synthesis	5.17 (HTC116)	Topoisomerase inhibition	[171]
**29**	Isolation	2.80 (HL-60)	Caspase activation and PG-E2 inhibition	[172]
**30**		3.40 (HL-60)		
**31**		3.10 (HL-60)		
**32**	Synthesis	4.50 (MDA-MB-231)	-	[173]
**33**	Synthesis	2.10 (A549)	Protein kinase inhibition	[174]
**34**	Synthesis	3.25 (HepG2)	-	[175]
**35**	Isolation	0.73 (CNE-2)	Antiproliferative induction	[176]
**36**		8.61 (NCI/ADR-RES)		
**37**	Isolation	1.30 (HL-60)	Antiproliferative induction	[178]
**38**	Isolation	3.35 (CNE-1)	Apoptosis induction	[67]
**39**	Isolation	0.87 (HL-60)	-	[179]
**40**		4.66 (PC-3)		
**41**	Synthesis	18.6 (HepG2)	Caspase activation	[18]
**42**	Synthesis	1.90 (K562)	-	[157]
**43**	Synthesis	3.90 (MDA-MB-468)	-	[182]
**44**	Synthesis	3.60 (A375-C5)	Apoptosis induction	[183]
**45**	Synthesis	>165 (HT-29)	-	[184]

## Data Availability

Data sharing not applicable.
